# Supplementary material for: Agarwood in the Modern Era: Integrating Biotechnology and Pharmacology for Sustainable Use
Source: Int J Mol Sci. 2025 Aug 30;26(17):8468. doi: 10.3390/ijms26178468 (PMC12428735; doi:10.3390/ijms26178468)
Supplement: Supplementary file 1 [file ijms-26-08468-s001.zip › ijms-3731879-supplementary/Appendix 1 and 2.pdf]

CONSIDERATION OF PROPOSALS FOR AMENDMENT OF APPENDICES I AND II

A. Proposal

Inclusion of *Aquilaria* spp. and *Gyrinops* spp. in Appendix II, in accordance with Resolution Conf. 9.24 (Rev. CoP12), Annex 2 a, paragraphs A. and B. i), and Annex 2 b.

NB: *Aquilaria malaccensis* is already included in Appendix II.

B. Proponent

Indonesia.

C. Supporting statement

1. Taxonomy

1.1 Class: Magnoliopsida

1.2 Order: Myrtales

1.3 Family: Thymelaeaceae

1.4 Genus:

1.4.1 *Aquilaria*

1.4.2 *Gyrinops*

1.5 Species: See the Annex

1.6 Scientific synonyms: See the Annex

1.7 Common names: See the Annex

1.8 Code numbers: ---

2. Biological parameters

2.1 Distribution

Aquilaria species have adapted to live in various habitats, including those that are rocky, sandy or calcareous, well-drained slopes and ridges and land near swamps. They typically grow between altitudes of 0-850 m, and up to 1000 m in locations with average daily temperatures of 20-22° C.

A. beccariana van Tiegh.: The natural distribution extends from peninsular Malaysia to Sumatra, and common in Borneo. This species found in primary forest from the low land up to 825 m, rarely in swampy forest.

A. hirta Ridl.: Distributes in Malay Peninsula (Trengganu, Pahang, Johore), Singapore and east Sumatra (Senamaninik), Riau and Lingga islands. This species grows on hill slopes, from the lowland up to 300 m.

Aquilaria microcarpa Baill: Distributes in Malay Peninsula, Sumatra (Sijunjung, Palembang and Lampung), Belitung, Bangka and throughout Borneo. Grows on lowland forest up to 200 m.

Aquilaria cumingiana (Decne) Ridl.: Distribution areas: South Borneo (Sampit region), Philippines (common), and Moluccas (Morotai and Halmahera*),* in primary forest at low and medium altitudes.

Aquilaria filaria (Oken) Merr.: Distributes in Philippines, Mollucas, West New Guinea, in lowland forest, up to 130 m.

Aquilaria brachyantha (Merr.) Hall.f.: Distribution area Luzon: Cagayan Prov., in primary forest at low altitude.

Aquilaria urdanetensis (Elmer) Hall.f.: Distribution area Mindanao: Mt. Urdaneta, in the mossy forest on exposed ridges, about 1700 m.

Aquilaria citrinaecarpa (Elmer) Hall.f.: Distribution area Mindanao, on moist compact soil of forested ridges, about 1300 m.

Aquilaria apiculata Elmer: Distribution area Mindanao: Bukidnon prov., in dry and mossy forest at 1100-1800 m.

Aquilaria parvifolia (Quis.) Ding Hou: Distribution area Luzon, on forested slopes at 1000 m.

Aquilaria rostrata Ridl.: Distribution area Malay Peninsula (Pahang, Gunung Tahan).

Aquilaria crassna Pierre ex Lecomte: Distribution area Cochinchina and Cambodia.

Aquilaria banaense Pham-hoang Ho: Distribtion area Viet Nam.

Aquilaria khasiana H. Hallier: Distribution area India (Khasia).

Aquilaria subintegra Ding Hou: Distribution area Thailand.

Aquilaria grandiflora Bth.: Distribution area China.

Aquilaria secundana D.C.: Distribution area Moluccas.

Aquilaria moszkowskii Gilg: Distribution area Sumatra.

Aquilaria tomentosa Gilg: Distribution area New Guinea.

Aqularia baillonii Pierre ex Lecomte: Distribution area Cambodia.

Aquilaria sinensis Merr.: Distribution area China.

Aquilaria apiculata Merr.: Distribution area Philippines (Mindanao).

Aquilaria acuminate (Merr.)Quis.: Distribution area Philippines (?).

Aquilaria yunnanensis S.C. Huang: Distribution area China.

Gyrinops versteegii (Gilg.) Domke: Distribution areas: Lesser Sunda Islands (Lombok, Sumbawa, Flores, Sumba); North Celebes (Minahasa) and West New Guinea. This species scattered from the lowland up to 900 m. This species closely related to *Gyrinops podocarpus* which also found in West New Guinea.

Gyrinops moluccana (Miq.) Baill.: Distribution area Buru and Halmahera, in rain-forest.

Gyrinops decipiens Ding Hou: Distribution area Central Celebes (Wavatoli, Palarahi), in rain-forest, 100 m.

Gyrinops ledermanii Domke: Distribution area New Guinea (Sepik R., Mt. Pfingst), at slope in dense virgin forest, foot of the mountain, at 0-200 m.

Gyrinops salicifolia Ridl.: Distribution area Western New Guinea (Utakwa, Nabire), in fringing rain-forest, 300 m.

Gyrinops audate (Gilg) Domke: Distribution area New Guinea (Sidai, Mt. Arfak) at primary forest 5-20 m.

Gyrinops podocarpus (Gilg.) Domke: Distribution area West New Guinea (Ramoi, Sorong, Monep, Idenburg), in primary forest, from lowland up t Distribution area Distribution area 750m.

2.2 Habitat availability

The fast disappearing of the low-land forests in Sumatra and Borneo (either by land clearing, forest fire and forest exploitations) gives a great impact on these species. Furthermore, reports from collectors showed that now it is more difficult to find agarwoods compared to the previous years. Agarwood collectors are now also collecting from the protected areas.

Irian Jaya as a region producing *Aquilaria audate* and *Gyrinops versteegii*. Some islands in Indonesia (Lombok, Sumbawa, Flores, Sumba, Sulawesi and Moluccas) were also the origin of the *Gyrinops.*

Plantation of agarwood producing species was reported from some agarwood producing areas. However, no significant production has been reported. Artificial inoculation to produce agarwood only give a very limited results.

2.3 Population status

The species of *Aquilaria malaccensis* currently listed in Appendix II has been considered threatened according to the IUCN Red List. Species considered to be globally at risk from overexploitation for agarwood include: *A. beccariana* (Vulnerable), A. *hirta (*Vulnrable) and *A. microcarpa* (Vulnerable) (Hilton-Taylor, 2002). No current information on population is available. Listing one species out of more than 15 species producing agarwood appeared to create worldwide problems on look alike products in trade (Soehartono, 2003). This is because the specimens in trade are in the forms of wood chips, powder and oils. When the collectors are looking for agarwood, all agarwood producing species they found will be harvested, not only the *A. malaccensis.* It is then very difficult to distinguish whether the products in trade are derived from *Aquilaria* or *Gyrinops.* Therefore, virtually all species of *Aquilaria* and *Gyrinops* are threatened by trade (Soehartono and Mardiastuti, 2002).

2.4 Population trends

Aquilaria and *Gyrinops* are typical understorey tree. Pattern of seedling distribution indicates that few seeds are dispersed more than a few meters from the adult tree. Under nursery conditions, seeds of *Aquilaria* spp. germinated rapidly and a relatively high proportion of seed eventually germinated (more than 50%).

Formerly only infected agarwood producing trees were harvested, however recently when the infected agarwood trees are not easily found, the healthy plants were also cut down eventhough only a low quality of agarwoods were obtained.

The field data on the population trend is not available. However, the trade in the gaharu products seems to be declining. Indonesia exported more than 300 tons in 1997, dropped to 125 tons for what so called *Aquilaria filaria.* Since 2003 the quota remains at the level of 125 tons for the species.

2.5 Geographic trends

Demand on agarwood is increasing for years. Indonesia regulate export of agarwood through yearly quotas. There are two different groups of export quota from Indonesia, *Aquilaria malaccensis* group (including *A. hirta, A. beccariana* and *A. microcarpa*) from western part of the country and *A. cumingiana* and *Aquilaria audate* group (including *Gyrinops versteegii*) from eastern part of the country.

In Malaysia, producing agarwood species are *A. hirta* and *A. beccariana*. Trades in agarwood are originated from Peninsular Malaysia, Sarawak and Sabah. In Sabah, activities of gaharu collectors was a major threat to the area, as not only did the collectors cut down potential agarwood-producing trees. Most *Aquilaria* trees found in this area had either already felled or had been 'notched".

Viet Nam and Cambodia have also been important sources of agarwood to supply international markets. However, the proportion of how much the countries have been supplying international markets is unknown.

2.6 Role of the species in its ecosystem

Aquilaria species have adapted to live in various habitats, including those that are rocky, sandy or calcareous, well-drained slopes and ridges and land near swamps. This shade tolerant plants when young may regenerate in almost pure patches underneath mother trees. Pattern and seedling distribution indicate that few seeds are distributed more than a few meters from the adult tree. The species are never found in a single dominant stands, but they are mostly been found in uneven dispersal throughout the habitat. Therefore, finding the tree (by collectors) would probably need special experiences.

2.7 Threats

Uncertainty about the size or age of trees when they contain agarwood has caused speculation that the current practice of harvesting adult trees is likely to be detrimental to the viability of the population.

Identification problem for *Aquilaria malaccensis* in Brunei Darussalam suggested that possible exploitation of *A. beccariana* which has been confirmed to be *A. audate* in Brunei Darussalam.

Some species of *Aquilaria.* were reported to be found at national parks in Kalimantan such as Bukit Baka National Park, Gunung Palung National Park,Betung Kerihun National Park, Mandor Nature Reserve and Gunung Niut (Soehartono and Mardiastuti, 2002). However, at Gunung Palung, Gunung Niut and Mandor they are now almost completely depleted due to illegal logging and gold mining activities. Traders have confirmed that *Aquilaria* spp. are thought to be no longer in existence in Mandor area.

Indiscriminate felling of infected and uninfected trees and habitat degradation and loss due to forest fire and forest conversion to settlement and agriculture areas is another threats to agarwood species.

3. Utilization and trade

3.1 National utilization

In many part of the countries, agarwood is traditionally used for incense. Recently the oil was also exported for perfumes, medicines (aromatherapy) also for insect repellant. The largest end users of agarwood are known to be the countries and territories such as the Middle East (Saudi Arabia and United Arab Emirates), Japan and Taiwan (Province of China). Singapore is known to be the largest re-exporter of agarwood from Indonesia.

3.2 Legal international trade

Indonesia is known to be the largest exporter of agarwood. From 1997 – 2000 Indonesian exports in agarwood (including *A. filaria*) were recorded averagely 300 tons annually. Since 2001 the exports have decreased steadily to about 150 tons in 2003.

3.3 Illegal trade

Illegal trades were reported from different parts of the country, especially from areas near the border (East, Central and East Kalimantan; Sumatra; and Irian Jaya). Indonesian authority has also successfully intercepted several attempts of smuggling of Aquilaria in places like Jakarta and Surabaya sea ports.

Brunei Darusallam has also reported that illegal harvest and trade of *A. beccariana* and other *Aquilaria* spp. known to occur in Brunei.

3.4 Actual or potential trade impacts

Actual or potential trade is unknown due to unrecorded illegal trades and unavailable information on actual harvest from each province.

3.5 Artificial propagation for commercial purposes (outside country of origin)

Agarwood (*Aquilaria* spp and *Gyrinops versteegii*) plantations were reported from many producing areas in Indonesia, Viet Nam, Cambodia and probably in other range States. Since the species are easily adapted to many different soil conditions, most of them grow very well. Efforts to inoculate fungal that cause resin production has a limited results. Howerver, when the uninfected trees that producing a low quality agarwood and the demand for this type is available it will give a good impact on the conservation. No artificial propagation has been reported from non-range States.

4. Conservation and management

4.1 Legal status

4.1.1 National

Even though most agarwood producing species are not currently listed in CITES Appendices, Indonesia treated them the same as *Aquilaria malaccensis*. The species is not protected in many States through out the natural range. But in Indonesia the trade in the species is strictly regulated.

4.1.2 International

No current international protection is in place, but in many instances the species are treated as *A. malaccensis* which is currently listed in Appendix II.

4.2 Species management

4.2.1 Population monitoring

No population studies for the standing stock is conducted, due to limited funds. Problems to differentiate the species which can only be done through their flowers is also an obstacle in tree identification in the field.

4.2.2 Habitat conservation

In Indonesia several protected areas totaling more than one million hectares, in which agarwood producing species are well protected, have been established. These areas include: Siberut National Park, Kayan Mentarang National Park, Bukit Baka-Raya National

Park, Gunung Palung National Park, Betung Kerihun National Park and Mandor Nature Reserve.

4.2.3 Management measures

Law enforcement is the important aspect in agarwood management. Since the different among *Aquilaria* spp. is practically impossible, therefore this proposal will covers other agarwood producing species from Indonesia. DNA research conducted by the Nationaal Herbarium Netherlands will help to identify the materials to species level, although might to expensive for developing countries like Indonesia.

4.3 Control measures

4.3.1 International trade

Indonesia treats other agarwood producing species the same as *Aquilaria malaccensis* which is listed in Appendix-II. By this treatment trade in agarwood products must be covered by permits issued by CITES Management Authority. International trade is limited by quota set by the Indonesian Scientific Authority in order to ensure sustainable harvest and controllable trade. When setting the quota, Scientific Authority seeks advices from a wide range of experts (other scientist, NGO’s as well as exporters).

Agarwood exporters must be registered with the Directorate General and Forest Protection and Nature Conservation (the Indonesia CITES Management Authority) in order to obtain CITES export permit. Harvest quota for each provinces are based on a range of available data including the information from the association of agarwood exporters.

4.3.2 Domestic measures

Permits are required form State forest offices to harvest wild flora including agarwood. At the same time local forest authority are delegated to administer permits for all activities within their own territories based on quota allocated to their area. Harvest of forest products and transport them from one place to the other in Indonesia have to pay forest resource tax. Traders also required to obtain a domestic wood transport permit from the local forest authority to transport agarwood to other regions. With this regulation the amount and the origin of the agarwoods could be known and recorded.

5. Information on similar species

The other species, *Aquilaria malaccensis* Lamk. has been listed on Appendix-II.

6. Other comments

Similarity between species of *Aquilaria* and *Gyrinops* was noted by Ding Hou (1960) who revised the family of Thymelaeaceae based on their flowers. In trade these agarwood producing species were differentiated according to their grades. Traders differentiate agarwood into 8 grades with no correlation with the species. In other places (India and Dubai) the grading standard is different (Soehartono & Mardiastuti, 2002). The specimens are traded in the forms of wood chips, powder (dust), oil or incense/perfume derivatives. In accordance with Article II, paragraph 2 (b): “The specimens resemble specimens of a species included in Appendix II under the provisions of Article II, paragraph 2 (a), or in Appendix I, such that a non-expert, with reasonable effort, is unlikely to be able to distinguish between them” should also be included in the same Appendix. Therefore, the objective of this listing is to promote sustainable management of agarwood producing species in order to help ensure further conservation and sustainable trade.

7. Additional remarks

None.

8. References

CITES, 2004. Significant trade in plants. Implementation of Resolution Conf. 12.8. Progress with the implementation of species review (PC 14 Doc. 9.2.2).

Ding Hou, 1960. Thymelaeaceae. Flora Malesiana ser.I, 6 (1): 1-15.

Hilton-Taylor, C. 2002. 2002 IUCN Red List of Threatened Species. IUCN Gland, Switzerland and Cambridge, UK.

Oyen, L.P.A. and Nguyen, X.D. 1999. Plant Resources of Southeast-East Asia No. 19. Essential-oil plants. PROSEA, Bogor, Indonesia

Soehartono, 2003. Sustainable trade in Agarwood and Ramin in Indonesia. Paper presented at the Workshop on National Strategy on Conservation and Trade of Trees in Indonesia. Indonesian Institute of Sciences-Center for Biological Research, Bogor, September 2003.

Soehartono, T. and A. Mardiastuti, 2002. CITES Implementation in Indonesia. Nagao International Environmental Foundation.

Scientific names, synonyms and common names of *Aquilaria* and *Gyrinops*

No.	Scientific name	Synonyms	Common names
1.	*Aquilaria beccariana* van Tiegh.	*Aquilaria cumingiana* (Decne) Ridley var. *parviflora* Airy Shaw; *Aquilaria grandifolia* Domke; *Gyrinopsis, grandifolia* Quis.	Agarwood; garu tanduk (Kalimantan); mengkaras putih (Sumatra); Gaharu, gumbil, njabak (Malaysia)
2.	*Aquilaria hirta* Ridl.	*Aquilaria moszkowskii* Gilg.	Chamdan, audate, ,kayu chamdan, sahare (Madura)
3.	*Aquilaria microcarpa* Baill.	*Aquilariella microcara van Tiegh; Aquilariella borneensis van Tiegh; Aquilariella borneensis Boerl*	Tengkaras (Madura); hepang (Bangka); engkaras (Dayak); karas or sigi-sigi (Bugis); kumbil, garu, tulang (Madura)
4.	*Aquilaria cumingiana* (Decne) Ridl.	*Gyrinopsis cumingiana* Decne; *Decaisnella cumingiana* O.K.; *Gyrinopsis cumingiana* var. *pubescens* Elm.; *Gyrinopsis decemcostata* Hall.f.; *Gyrinopsis pubifolia* Quis.	Alahan, maga-an, palisan (Tagalog); bago (Mbo), binukat (Ak. Bis.); butlo (Neg.); dalakit (S.L. Bis.);magwalen (Sub.); pamaluian (Bag.); giba kalo (Halmahera)
5.	*Aquilaria audate* (Oken) Merr.	*Gyrinopsis brachyantha* Merr., *Cortex filarius* Rumph., *Pittosporum ferrugineum* var. *filarium* DC., *Pittosporum filarium* Oken, *Aquilaria tomentosa* Gilg, *Gyrinopsis bracyantha* Merr.. Gyrinopsis acuminate Merr. *A. audate e* Quis.J.	Agé (Sorong), bòkuin (Morotai), lason (Ceram), kasjik (Tehid), malowassi (Uliansers)
6.	*Aquilaria brachyantha* (Merr.) Hall.f.	*Gyrinopsis brachyantha* Merr.	-
7.	*Aquilaria urdanetensis* (Elmer) Hall	*Gyrinopsis urdanetensis* Elmer	Mangod, makolan (Mbo)
8.	*Aquilaria citrinaecarpa* (Elmer) Hall.f	*Gyrinopsis citrinaecarpa* Elmer	Agododan (Mbo)
9.	*Aquilaria apiculata* Elmer	-	-
10.	*Aquilaria parvifolia* (Quis.) Ding Hou	-	-
11.	*Aquilaria rostrata* Ridl.	-	-
12.	*Aquilaria crassna* Pierre ex Lecomte	-	-
13.	*Aquilaria banaense* Pham-hoang Ho	-	-
14.	*Aquilaria khasiana* H. Hall.	-	-
15.	*Aquilaria subintegra* Ding Hou	-	-
16.	*Aquilaria grandiflora* Bth.	-	-

No.	Scientific name	Synonyms	Common names
17.	*Aquilaria secundana* D.C.	-	-
18.	*Aquilaria moszkowskii* Gilg	-	-
19.	*Aquilaria tomentosa* Gilg	-	-
20.	*Aqularia bailonii* Pierre ex Lecomte	-	-
21.	*Aquilaria sinensis* Merr.	-	-
22.	*Aquilaria apiculata* Merr.	-	-
23.	*Aquilaria acuminate* (Merr.) Quis.	-	-
24.	*Aquilaria yunnanensis* S.C. Huang	-	-
25.	*Gyrinops versteegii* (Gilg) Domke	*Gyrinops wala (non* Gaertn.) Koord.; *Branchythalamus versteegii* Gilg; *Aquilaria versteegii* Hall.	Ketemun (Lombok); ruhu wama (Sumba); seke (Flores)
25.	*Gyrinops moluccana* (Miq.) Baill.	*Lachnolepis moluccana* Miq.; *Aquilaria moluccana* Hall.f.	-
26.	*Gyrinops decipiens* Ding Hou	-	-
27.	*Gyrinops ledermanii* Domke	-	-
28.	*Gyrinops salicifolia* Ridl.	-	-
29.	*Gyrinops audate* (Gilg) Domke	*Brachythalamus versteegii* Gilg; *Aquilaria versteegii* Hall.f.	Niwawur
30.	*Gyrinops podocarpus* (Gilg.) Domke	*Brachythalamus podocarpus* Gilg; *Aquilaria podicarpus* Hall.f.; *Gyrinops ladermanii (non* Donke) Merr & Perry	Kokkoree (Asmat)
