# Supplementary material for: Agarwood in the Modern Era: Integrating Biotechnology and Pharmacology for Sustainable Use
Source: Int J Mol Sci. 2025 Aug 30;26(17):8468. doi: 10.3390/ijms26178468 (PMC12428735; doi:10.3390/ijms26178468)
Supplement: Supplementary file 1 [file ijms-26-08468-s001.zip › ijms-3731879-supplementary/E-Appendices-2025-02-07.pdf]

Appendices I, II and III

valid from 7 February 2025

Interpretation

1. Species included in these Appendices are referred to:

   a) by the name of the species; or
   b) as being all of the species included in a higher taxon or designated part thereof.

2. The abbreviation "spp." is used to denote all species of a higher taxon.

3. Other references to taxa higher than species are for the purposes of information or classification only. The common names included after the scientific names of families are for reference only. They are intended to indicate the species within the family concerned that are included in the Appendices. In most cases this is not all of the species within the family.

4. The following abbreviations are used for plant taxa below the level of species:

   a) "ssp." is used to denote subspecies; and
   b) "var(s)." is used to denote variety (varieties).

5. As none of the species or higher taxa of FLORA included in Appendix I is annotated to the effect that its hybrids shall be treated in accordance with the provisions of Article III of the Convention, this means that artificially propagated hybrids produced from one or more of these species or taxa may be traded with a certificate of artificial propagation, and that seeds and pollen (including pollinia), cut flowers, seedling or tissue cultures obtained *in vitro* transported in sterile containers of these hybrids are not subject to the provisions of the Convention.

6. The names of the countries in parentheses placed against the names of species in Appendix III are those of the Parties submitting these species for inclusion in this Appendix.

7. When a species is included in Appendix I, II or III, the whole, live or dead, animal or plant is always included. In addition, all parts and derivatives thereof are also included in the same Appendix unless, for animal species listed in Appendix III and plant species listed in Appendix II or III, the species is annotated with the symbol # followed by a number to indicate that only specific parts and derivatives are included. The symbol # followed by a number placed against the name of a species or higher taxon included in Appendix II or III refers to a footnote that indicates the parts or derivatives of animals or plants that are designated as 'specimens' subject to the provisions of the Convention in accordance with Article I, paragraph (b), subparagraph (ii) or (iii).

8. The terms and expressions below, used in annotations in these Appendices, are defined as follows:

   <u>Extract</u>

   Any substance obtained directly from plant material by physical or chemical means regardless of the manufacturing process. An extract may be solid (e.g. crystals, resin, fine or coarse particles), semi-solid (e.g. gums, waxes) or liquid (e.g. solutions, tinctures, oil and essential oils).

   <u>Finished musical instruments</u>

   A musical instrument (as referenced by the Harmonized System of the World Customs Organization, Chapter 92; musical instruments, parts and accessories of such articles) that is ready to play or needs only the installation of parts to make it playable. This term includes antique instruments (as defined by the Harmonized System codes 97.05 and 97.06; Works of art, collectors' pieces and antiques).

Finished musical instrument accessories

A musical instrument accessory (as referenced by the Harmonized System of the World Customs Organization, Chapter 92; musical instruments, parts and accessories of such articles) that is separate from the musical instrument, and is specifically designed or shaped to be used explicitly in association with an instrument, and that requires no further modification to be used.

Finished musical instrument parts

A part (as referenced by the Harmonized System of the World Customs Organization, Chapter 92; musical instruments, parts and accessories of such articles) of a musical instrument that is ready to install and is specifically designed and shaped to be used ex[1]plicitly in association with the instrument to make it playable.

Finished products packaged and ready for retail trade

Products, shipped singly or in bulk, requiring no further processing, packaged, labelled for final use or the retail trade in a state fit for being sold to or used by the general public.

Powder

A dry, solid substance in the form of fine or coarse particles.

Shipment

Cargo transported under the terms of a single bill of lading or air waybill, irrespective of the quantity or number of containers or packages; or pieces worn, carried or included in personal baggage.

Ten (10) kg per shipment

For the term "10 kg per shipment", the 10 kg limit should be interpreted as referring to the weight of wood of each individual annotated species of genus Dalbergia *or* Guibourtia *present in the items in the shipment. The 10 kg limit is to be assessed only against the individual weights of the portions of wood of each individual annotated species contained in each item of the shipment, and not against the total weight of the shipment. The total weights present of each individual annotated species are considered individually to determine whether a CITES permit or certificate is required for each individual annotated species, and weights of different individual annotated species are not added together for this purpose.*

Transformed wood

Defined by Harmonized System code 44.09: Wood (including strips, friezes for parquet flooring, not assembled), continuously shaped (tongued, grooved, rebated, chamfered, V-jointed, beaded, moulded, rounded or the like) along any of its edges, ends or faces, whether or not planed, sanded or end-jointed.

Woodchips

Wood that has been reduced to small pieces.

1

	Appendices I	II	III
**FAUNA (ANIMALS)**<br>**PHYLUM CHORDATA**<br>**CLASS MAMMALIA (MAMMALS)**			
ARTIODACTYLA			
ANTILOCAPRIDAE Pronghorns			
	Antilocapra americana (Only the population of Mexico; no other population is included in the Appendices)		
BOVIDAE Antelopes, cattle, duikers, gazelles, goats, sheep, etc.			
	Addax nasomaculatus		
		Ammotragus lervia	
			Antilope cervicapra (Nepal, Pakistan)
	Bos gaurus {Excludes the domesticated form, which is referenced as *Bos frontalis*, and is not subject to the provisions of the Convention}		
	Bos mutus {Excludes the domesticated form, which is referenced as *Bos grunniens*, and is not subject to the provisions of the Convention}		
	Bos sauveli		
			Boselaphus tragocamelus (Pakistan)
			Bubalus arnee {Excludes the domesticated form, which is referenced as *Bubalus bubalis* and is not subject to the provisions of the Convention} (Nepal)
	Bubalus depressicornis		
	Bubalus mindorensis		
	Bubalus quarlesi		
		Budorcas taxicolor	
		Capra caucasica	
	Capra falconeri		

	Appendices I	II	III
			Capra hircus aegagrus [A1] (Pakistan)
			Capra sibirica (Pakistan)
	Capricornis milneedwardsii		
	Capricornis rubidus		
	Capricornis sumatraensis		
	Capricornis thar		
		Cephalophus brookei	
		Cephalophus dorsalis	
	Cephalophus jentinki		
		Cephalophus ogilbyi	
		Cephalophus silvicultor	
		Cephalophus zebra	
		Damaliscus pygargus pygargus	
			Gazella bennettii (Pakistan)
	Gazella cuvieri		
			Gazella dorcas (Algeria, Tunisia)
	Gazella leptoceros		
	Hippotragus niger variani		
		Kobus leche	
	Naemorhedus baileyi		
	Naemorhedus caudatus		
	Naemorhedus goral		
	Naemorhedus griseus		
	Nanger dama		
	Oryx dammah		
	Oryx leucoryx		
		Ovis ammon	
		Ovis arabica	
		Ovis bochariensis	
		Ovis canadensis (Only the population of Mexico; no other population is included in the Appendices)	

[A1] Specimens of the domesticated form are not subject to the provisions of the Convention.

	Appendices I	II	III
		Ovis collium	
		Ovis cycloceros	
		Ovis darwini	
	Ovis gmelini (Only the population of Cyprus; no other population is included in the Appendices)		
	Ovis hodgsoni		
		Ovis jubata	
		Ovis karelini	
	Ovis nigrimontana		
		Ovis polii	
		Ovis punjabiensis	
		Ovis severtzovi	
	Ovis vignei		
	Pantholops hodgsonii		
		Philantomba maxwelli	
		Philantomba monticola	
			Pseudois nayaur (Pakistan)
	Pseudoryx nghetinhensis		
		Rupicapra pyrenaica ornata	
		Saiga borealis [A2]	
		Saiga tatarica [A2]	
			Tetracerus quadricornis (Nepal)

[A2] A zero export quota for wild specimens traded for commercial purposes.

	Appendices I	II	III
CAMELIDAE Guanaco, vicuna			
		Lama guanicoe	
	Vicugna vicugna (Except the populations of: Argentina [populations of the Provinces of Catamarca, Jujuy and Salta, and semi-captive populations of the Provinces of Catamarca, Jujuy, La Rioja, Salta and San Juan], Chile [populations of the region of Arica and Parinacota and of the region of Tarapacá], Ecuador, Peru and the Plurinational State of Bolivia, which are included in Appendix II)	***Vicugna vicugna*** [A3] (Only the populations of Argentina [populations of the Provinces of Catamarca, Jujuy and Salta, and semi-captive populations of the Provinces of Catamarca, Jujuy, La Rioja, Salta and San Juan], Chile [populations of the region of Arica and Parinacota and of the region of Tarapacá], Ecuador, Peru and the Plurinational State of Bolivia; all other populations are included in Appendix I)	

[A3] For the exclusive purpose of allowing international trade in fibre from vicuñas (*Vicugna vicugna*) and their derivative products, only if the fibre comes from the shearing of live vicuñas. Trade in products derived from the fibre may only take place in accordance with the following provisions:

a) Any person or entity processing vicuña fibre to manufacture cloth and garments must request authorization from the relevant authorities of the country of origin (Countries of origin: The countries where the species occurs, that is, Argentina, Bolivia, Chile, Ecuador and Peru) to use the "vicuña country of origin" wording, mark or logo adopted by the range States of the species that are signatories to the Convention for the Conservation and Management of the Vicuña.

b) Marketed cloth or garments must be marked or identified in accordance with the following provisions:

   i) For international trade in <u>cloth</u> made from live-sheared vicuña fibre, whether the cloth was produced within or outside of the range States of the species, the wording, mark or logo must be used so that the country of origin can be identified. The VICUÑA [COUNTRY OF ORIGIN] wording, mark or logo has the format as detailed below:

   VICUÑA [PAÍS DE ORIGEN]

   This wording, mark or logo must appear on the reverse side of the cloth. In addition, the selvages of the cloth must bear the words VICUÑA [COUNTRY OF ORIGIN].

   ii) For international trade in <u>garments</u> made from live-sheared vicuña fibre, whether the garments were produced within or outside of the range States of the species, the wording, mark or logo indicated in paragraph b) i) must be used. This wording, mark or logo must appear on a label on the garment itself. If the garments are produced outside of the country of origin, the name of the country where the garment was produced should also be indicated, in addition to the wording, mark or logo referred to in paragraph b) i).

c) For international trade in handicraft products made from live-sheared vicuña fibre produced within the range States of the species, the VICUÑA [COUNTRY OF ORIGIN] - ARTESANÍA wording, mark or logo must be used as detailed below:

   VICUÑA [PAÍS DE ORIGEN] - ARTESANÍA

d) If live-sheared vicuña fibre from various countries of origin is used for the production of cloth and garments, the wording, mark or logo of each of the countries of origin of the fibre must be indicated, as detailed in paragraphs b) i) and ii).

e) All other specimens shall be deemed to be specimens of species listed in Appendix I and the trade in them shall be regulated accordingly.

	Appendices I	II	III
CERVIDAE Deer, huemuls, muntjacs, pudus			
	Axis calamianensis		
	Axis kuhlii		
			Axis porcinus (Except the subspecies included in Appendix I) (Pakistan)
	Axis porcinus annamiticus		
	Blastocerus dichotomus		
		Cervus elaphus bactrianus	
			Cervus elaphus barbarus (Algeria, Tunisia)
	Cervus elaphus hanglu		
	Dama dama mesopotamica		
	***Hippocamelus* spp.**		
			Mazama temama cerasina (Guatemala)
	Muntiacus crinifrons		
	Muntiacus vuquangensis		
			Odocoileus virginianus mayensis (Guatemala)
	Ozotoceros bezoarticus		
		Pudu mephistophiles	
	Pudu puda		
	Rucervus duvaucelii		
	Rucervus eldii		
GIRAFFIDAE Giraffe			
		Giraffa camelopardalis	
HIPPOPOTAMIDAE Hippopotamuses			
		Hexaprotodon liberiensis	
		Hippopotamus amphibius	
MOSCHIDAE Musk deer			
	***Moschus* spp.** (Only the populations of Afghanistan, Bhutan, India, Myanmar, Nepal and Pakistan; all other populations are included in Appendix II)	***Moschus* spp.** (Except the populations of Afghanistan, Bhutan, India, Myanmar, Nepal and Pakistan, which are included in Appendix I)	

	Appendices I	II	III
SUIDAE Babirusa, hog			
	Babyrousa babyrussa<br>***Babyrousa bolabatuensis***<br>***Babyrousa celebensis***<br>***Babyrousa togeanensis***<br>***Sus salvanius***		
TAYASSUIDAE Peccaries			
	Catagonus wagneri	**TAYASSUIDAE spp.** (Except the species included in Appendix I and the populations of *Pecari tajacu* of Mexico and the United States of America, which are not included in the Appendices)	
CARNIVORA			
AILURIDAE Red panda			
	Ailurus fulgens		
CANIDAE Wild dogs, foxes, wolves			
			Canis aureus (India)
	Canis lupus (Only the populations of Bhutan, India, Nepal and Pakistan; all other populations are included in Appendix II) {Excludes the domesticated form and the dingo which are referenced as *Canis lupus familiaris* and *Canis lupus dingo*, respectively, which are not subject to the provisions of the Convention}	***Canis lupus*** (Except the populations of Bhutan, India, Nepal and Pakistan, which are included in Appendix I) {Excludes the domesticated form and the dingo which are referenced as *Canis lupus familiaris* and *Canis lupus dingo*, respectively, which are not subject to the provisions of the Convention}	
		Cerdocyon thous<br>***Chrysocyon brachyurus***<br>***Cuon alpinus***<br>***Lycalopex culpaeus***<br>***Lycalopex fulvipes***<br>***Lycalopex griseus***<br>***Lycalopex gymnocercus***	
	Speothos venaticus		
			Vulpes bengalensis (India)

	Appendices I	II	III
		Vulpes cana	
			Vulpes vulpes griffithi (India)<br>***Vulpes vulpes montana*** (India)<br>***Vulpes vulpes pusilla*** (India)
		Vulpes zerda	
EUPLERIDAE Fossa, falanouc, Malagasy civet			
		Cryptoprocta ferox<br>***Eupleres goudotii***<br>***Fossa fossana***	
FELIDAE Cats			
		**FELIDAE spp.** [A1] [A4] (Except the species included in Appendix I)	
	Acinonyx jubatus [A5]<br>***Caracal caracal*** (Only the population of Asia; all other populations are included in Appendix II)<br>***Catopuma temminckii***<br>***Felis nigripes***<br>***Herpailurus yagouaroundi*** (Only the populations of Central and North America; all other populations are included in Appendix II)<br>***Leopardus geoffroyi***<br>***Leopardus guttulus***<br>***Leopardus jacobita***<br>***Leopardus pardalis***<br>***Leopardus tigrinus***<br>***Leopardus wiedii***		

[A1] Specimens of the domesticated form are not subject to the provisions of the Convention.

[A4] For *Panthera leo* (African populations): a zero annual export quota is established for specimens of bones, bone pieces, bone products, claws, skeletons, skulls and teeth removed from the wild and traded for commercial purposes. Annual export quotas for trade in bones, bone pieces, bone products, claws, skeletons, skulls and teeth for commercial purposes, derived from captive breeding operations in South Africa, will be established and communicated annually to the CITES Secretariat.

[A5] Annual export quotas for live specimens and hunting trophies are granted as follows: Botswana: 5; Namibia: 150; Zimbabwe: 50. The trade in such specimens is subject to the provisions of Article III of the Convention.

	Appendices I	II	III
	Lynx pardinus<br>***Neofelis diardi***<br>***Neofelis nebulosa***<br>***Panthera leo*** (Only the populations of India; all other populations are included in Appendix II)<br>***Panthera onca***<br>***Panthera pardus***<br>***Panthera tigris***<br>***Panthera uncia***<br>***Pardofelis marmorata***<br>***Prionailurus bengalensis bengalensis*** (Only the populations of Bangladesh, India and Thailand; all other populations are included in Appendix II)<br>***Prionailurus planiceps***<br>***Prionailurus rubiginosus*** (Only the population of India; all other populations are included in Appendix II)<br>***Puma concolor*** (Only the populations of Costa Rica and Panama; all other populations are included in Appendix II)		
HERPESTIDAE Mongooses			
			Herpestes edwardsi (India, Pakistan)<br>***Herpestes fuscus*** (India)<br>***Herpestes javanicus*** (Pakistan)<br>***Herpestes javanicus auropunctatus*** (India)<br>***Herpestes smithii*** (India)<br>***Herpestes urva*** (India)<br>***Herpestes vitticollis*** (India)
HYAENIDAE Aardwolf, hyena			
			Hyaena hyaena (Pakistan)<br>***Proteles cristata*** (Botswana)

	Appendices I	II	III
MEPHITIDAE Humboldt's hog-nosed skunk			
		Conepatus humboldtii	
MUSTELIDAE Badgers, martens, otters, weasels, etc.			
LUTRINAE Otters			
	Aonyx capensis microdon (Only the populations of Cameroon and Nigeria; all other populations are included in Appendix II)<br>***Aonyx cinereus***<br>***Enhydra lutris nereis***<br>***Lontra felina***<br>***Lontra longicaudis***<br>***Lontra provocax***<br>***Lutra lutra***<br>***Lutra nippon***<br>***Lutrogale perspicillata***<br>***Pteronura brasiliensis***	**LUTRINAE spp.** (Except the species included in Appendix I)	
MUSTELINAE Grisons, honey badgers, martens, tayra, weasels			
	Mustela nigripes		***Eira barbara*** (Honduras)<br>***Martes flavigula*** (India)<br>***Martes foina intermedia*** (India)<br>***Martes gwatkinsii*** (India)<br>***Mellivora capensis*** (Botswana)<br>***Mustela altaica*** (India)<br>***Mustela erminea ferghanae*** (India)<br>***Mustela kathiah*** (India)<br>***Mustela sibirica*** (India)
ODOBENIDAE Walrus			
			Odobenus rosmarus (Canada)

	Appendices<br>I	 II	 III
OTARIIDAE Fur seals, sealions			
	Arctocephalus townsendi	***Arctocephalus* spp.** (Except the species included in Appendix I)	
PHOCIDAE Seals			
	***Monachus* spp.**	***Mirounga leonina***	
PROCYONIDAE Coatis, kinkajou			
			Nasua narica (Honduras)<br>***Nasua nasua solitaria*** (Uruguay)<br>***Potos flavus*** (Honduras)
URSIDAE Bears, giant pandas			
	Ailuropoda melanoleuca<br>***Helarctos malayanus***<br>***Melursus ursinus***<br>***Tremarctos ornatus***<br>***Ursus arctos*** (Only the populations of Bhutan, China, Mexico and Mongolia; all other populations are included in Appendix II)<br>***Ursus arctos isabellinus***<br>***Ursus thibetanus***	**URSIDAE spp.** (Except the species included in Appendix I)	
VIVERRIDAE Binturong, civets, linsangs, otter-civet, palm civets			
	Prionodon pardicolor	***Cynogale bennettii***<br>***Hemigalus derbyanus***<br>***Prionodon linsang***	***Arctictis binturong*** (India)<br>***Civettictis civetta*** (Botswana)<br>***Paguma larvata*** (India)<br>***Paradoxurus hermaphroditus*** (India)<br>***Paradoxurus jerdoni*** (India)

	Appendices<br>I	II	III
			Viverra civettina (India)<br>***Viverra zibetha*** (India)<br>***Viverricula indica*** (India)
CETACEA Dolphins, porpoises, whales			
		**CETACEA spp.** [A6] (Except the species included in Appendix I)	
BALAENIDAE Bowhead whale, right whales			
	Balaena mysticetus<br>***Eubalaena* spp.**		
BALAENOPTERIDAE Fin whales, humpback whales, rorquals			
	Balaenoptera acutorostrata (Except the population of West Greenland, which is included in Appendix II)<br>***Balaenoptera bonaerensis***<br>***Balaenoptera borealis***<br>***Balaenoptera edeni***<br>***Balaenoptera musculus***<br>***Balaenoptera omurai***<br>***Balaenoptera physalus***<br>***Megaptera novaeangliae***		
DELPHINIDAE Dolphins			
	Orcaella brevirostris<br>***Orcaella heinsohni***<br>***Sotalia* spp.**<br>***Sousa* spp.**		
ESCHRICHTIIDAE Grey whale			
	Eschrichtius robustus		
INIIDAE River dolphin			
	Lipotes vexillifer		

[A6] For *Tursiops truncatus* (Black Sea population): a zero annual export quota has been established for live specimens removed from the wild and traded for primarily commercial purposes.

	Appendices I	II	III
NEOBALAENIDAE Pygmy right whale			
	Caperea marginata		
PHOCOENIDAE Porpoises			
	Neophocaena asiaeorientalis<br>***Neophocaena phocaenoides***<br>***Phocoena sinus***		
PHYSETERIDAE Sperm whale			
	Physeter macrocephalus		
PLATANISTIDAE River dolphins			
	***Platanista* spp.**		
ZIPHIIDAE Beaked whales, bottle-nosed whales			
	Berardius* spp.**<br>Hyperoodon* spp.**		
CHIROPTERA			
PHYLLOSTOMIDAE White-lined broad-nosed bats			
			Platyrrhinus lineatus (Uruguay)
PTEROPODIDAE Fruit bats, flying foxes			
	Acerodon jubatus<br>***Pteropus insularis***<br>***Pteropus loochoensis***<br>***Pteropus mariannus***<br>***Pteropus molossinus***<br>***Pteropus pelewensis***<br>***Pteropus pilosus***<br>***Pteropus samoensis***<br>***Pteropus tonganus***<br>***Pteropus ualanus***	***Acerodon* spp.** (Except the species included in Appendix I)<br>***Pteropus* spp.** (Except the species included in Appendix I and *Pteropus brunneus* which is not included in the Appendices)	

	Appendices I	II	III
	Pteropus yapensis		
CINGULATA			
DASYPODIDAE Armadillos			
	Priodontes maximus	***Chaetophractus nationi*** [A7]	***Cabassous tatouay*** (Uruguay)
DASYUROMORPHIA			
DASYURIDAE Dunnarts			
	Sminthopsis longicaudata<br>***Sminthopsis psammophila***		
DIPROTODONTIA			
MACROPODIDAE Kangaroos, wallabies			
	Lagorchestes hirsutus<br>***Lagostrophus fasciatus***<br>***Onychogalea fraenata***	***Dendrolagus inustus***<br>***Dendrolagus ursinus***	
PHALANGERIDAE Cuscuses			
		Phalanger intercastellanus<br>***Phalanger mimicus***<br>***Phalanger orientalis***<br>***Spilocuscus kraemeri***<br>***Spilocuscus maculatus***<br>***Spilocuscus papuensis***	
POTOROIDAE Rat-kangaroos			
	***Bettongia* spp.**		
VOMBATIDAE Northern hairy-nosed wombat			
	Lasiorhinus krefftii		

[A7] A zero annual export quota has been established. All specimens shall be deemed to be specimens of species included in Appendix I and the trade in them shall be regulated accordingly.

	Appendices I	II	III
LAGOMORPHA			
LEPORIDAE Rabbits			
	Caprolagus hispidus<br>***Romerolagus diazi***		
MONOTREMATA			
TACHYGLOSSIDAE Echidnas			
		Zaglossus **spp.**	
PERAMELEMORPHIA			
PERAMELIDAE Barred bandicoots			
	Perameles bougainville		
THYLACOMYIDAE Bilby			
	Macrotis lagotis		
PERISSODACTYLA			
EQUIDAE Horses, wild asses, zebras			
	Equus africanus {Excludes the domesticated form, which is referenced as *Equus asinus*, and is not subject to the provisions of the Convention}<br>***Equus grevyi***		
		Equus hemionus (Except the subspecies included in Appendix I)	
	Equus hemionus hemionus<br>***Equus hemionus khur***<br>***Equus hemionus luteus***		
		Equus kiang	
	Equus przewalskii		
		Equus zebra hartmannae<br>***Equus zebra zebra***	

	Appendices<br>I	 II	 III
RHINOCEROTIDAE Rhinoceroses			
	**RHINOCEROTIDAE spp.** (Except the subspecies included in Appendix II)	***Ceratotherium simum simum*** (Only the populations of Eswatini[A8], Namibia[A9] and South Africa[A8]; all other populations are included in Appendix I)	
TAPIRIDAE Tapirs			
	**TAPIRIDAE spp.** (Except the species included in Appendix II)	***Tapirus terrestris***	
PHOLIDOTA			
MANIDAE Pangolins			
	Manis crassicaudata<br>***Manis culionensis***<br>***Manis gigantea***<br>***Manis javanica***<br>***Manis pentadactyla***<br>***Manis temminckii***<br>***Manis tetradactyla***<br>***Manis tricuspis***	***Manis* spp.** (Except the species included in Appendix I)	
PILOSA			
BRADYPODIDAE Three-toed sloths			
		Bradypus pygmaeus<br>***Bradypus variegatus***	

[A8] The populations of Eswatini and South Africa of *Ceratotherium simum simum* are included in Appendix II for the exclusive purpose of allowing international trade in live animals to appropriate and acceptable destinations and hunting trophies. All other specimens shall be deemed to be specimens of species included in Appendix I and the trade in them shall be regulated accordingly.

[A9] The population of Namibia of *Ceratotherium simum simum* is included in Appendix II for the exclusive purpose of allowing international trade in live animals for *in-situ* conservation only, and only within the natural and historical range of *Ceratotherium simum* in Africa. All other specimens shall be deemed to be specimens of species included in Appendix I and the trade in them shall be regulated accordingly.

	Appendices I	II	III
MYRMECOPHAGIDAE Giant anteater, tamandua			
		Myrmecophaga tridactyla	***Tamandua mexicana*** (Guatemala)
PRIMATES Apes, monkeys			
		**PRIMATES spp.** (Except the species included in Appendix **I**)	
ATELIDAE Howler monkeys, spider monkeys			
	Alouatta palliata<br>***Alouatta pigra***<br>***Ateles geoffroyi frontatus***<br>***Ateles geoffroyi ornatus***<br>***Brachyteles arachnoides***<br>***Brachyteles hypoxanthus***<br>***Oreonax flavicauda***		
CEBIDAE Marmosets, tamarins, new-world monkeys			
	Callimico goeldii<br>***Callithrix aurita***<br>***Callithrix flaviceps***<br>***Leontopithecus* spp.**<br>***Saguinus bicolor***<br>***Saguinus geoffroyi***<br>***Saguinus leucopus***<br>***Saguinus martinsi***<br>***Saguinus oedipus***<br>***Saimiri oerstedii***		
CERCOPITHECIDAE Old-world monkeys			
	Cercocebus galeritus<br>***Cercopithecus diana***<br>***Cercopithecus roloway***<br>***Macaca silenus***<br>***Macaca sylvanus***<br>***Mandrillus leucophaeus***		

	Appendices I	II	III
	Mandrillus sphinx<br>***Nasalis larvatus***<br>***Piliocolobus kirkii***<br>***Piliocolobus rufomitratus***<br>***Presbytis potenziani***<br>***Pygathrix* spp.**<br>***Rhinopithecus* spp.**<br>***Semnopithecus ajax***<br>***Semnopithecus dussumieri***<br>***Semnopithecus entellus***<br>***Semnopithecus hector***<br>***Semnopithecus hypoleucos***<br>***Semnopithecus priam***<br>***Semnopithecus schistaceus***<br>***Simias concolor***<br>***Trachypithecus geei***<br>***Trachypithecus pileatus***<br>***Trachypithecus shortridgei***		
CHEIROGALEIDAE Dwarf lemurs			
	**CHEIROGALEIDAE spp.**		
DAUBENTONIIDAE Aye-aye			
	Daubentonia madagascariensis		
HOMINIDAE Apes, chimpanzees, gorillas, orang-utans			
	Gorilla beringei<br>***Gorilla gorilla***<br>***Pan* spp.**<br>***Pongo abelii***<br>***Pongo pygmaeus***<br>***Pongo tapanuliensis***		
HYLOBATIDAE Gibbons			
	**HYLOBATIDAE spp.**		
INDRIIDAE Indris, sifakas, woolly lemurs			
	**INDRIIDAE spp.**		

	Appendices I	II	III
LEMURIDAE Large lemurs			
	**LEMURIDAE spp.**		
LEPILEMURIDAE Sportive lemurs			
	**LEPILEMURIDAE spp.**		
LORISIDAE Lorises			
	***Nycticebus* spp.**		
PITHECIIDAE Sakis, uakaris			
	Cacajao* spp.**<br>Chiropotes albinasus***		

	Appendices I	II	III
PROBOSCIDEA			
ELEPHANTIDAE Elephants			
	Elephas maximus ***Loxodonta africana*** (Except the populations of Botswana, Namibia, South Africa and Zimbabwe, which are included in Appendix II subject to annotation A10)	***Loxodonta africana*** [A10] (Only the populations of Botswana, Namibia, South Africa and Zimbabwe; all other populations are included in Appendix I)	
RODENTIA			
CHINCHILLIDAE Chinchillas			
	***Chinchilla* spp.** [A1]		
CUNICULIDAE Pacas			
			Cuniculus paca (Honduras)
DASYPROCTIDAE Agoutis			
			Dasyprocta punctata (Honduras)

[A10] Populations of Botswana, Namibia, South Africa and Zimbabwe (listed in Appendix II):
For the exclusive purpose of allowing:

a) trade in hunting trophies for non-commercial purposes;
b) trade in live animals to appropriate and acceptable destinations, as defined in Resolution Conf. 11.20 (Rev. CoP18), for Botswana and Zimbabwe and for *in situ* conservation programmes for Namibia and South Africa;
c) trade in hides;
d) trade in hair;
e) trade in leather goods for commercial or non-commercial purposes for Botswana, Namibia and South Africa and for non-commercial purposes for Zimbabwe;
f) trade in individually marked and certified ekipas incorporated in finished jewellery for non-commercial purposes for Namibia and ivory carvings for non-commercial purposes for Zimbabwe;
g) trade in registered raw ivory (for Botswana, Namibia, South Africa and Zimbabwe, whole tusks and pieces) subject to the following:
   i) only registered government-owned stocks, originating in the State (excluding seized ivory and ivory of unknown origin);
   ii) only to trading partners that have been verified by the Secretariat, in consultation with the Standing Committee, to have sufficient national legislation and domestic trade controls to ensure that the imported ivory will not be re-exported and will be managed in accordance with all requirements of Resolution Conf. 10.10 (Rev. CoP18) concerning domestic manufacturing and trade;
   iii) not before the Secretariat has verified the prospective importing countries and the registered government-owned stocks;
   iv) raw ivory pursuant to the conditional sale of registered government-owned ivory stocks agreed at CoP12, which are 20,000 kg (Botswana), 10,000 kg (Namibia) and 30,000 kg (South Africa);
   v) in addition to the quantities agreed at CoP12, government-owned ivory from Botswana, Namibia, South Africa and Zimbabwe registered by 31 January 2007 and verified by the Secretariat may be traded and despatched, with the ivory in paragraph g) iv) above, in a single sale per destination under strict supervision of the Secretariat;
   vi) the proceeds of the trade are used exclusively for elephant conservation and community conservation and development programmes within or adjacent to the elephant range; and
   vii) the additional quantities specified in paragraph g) v) above shall be traded only after the Standing Committee has agreed that the above conditions have been met; and
h) no further proposals to allow trade in elephant ivory from populations already in Appendix II shall be submitted to the Conference of the Parties for the period from CoP14 and ending nine years from the date of the single sale of ivory that is to take place in accordance with provisions in paragraphs g) i), g) ii), g) iii), g) vi) and g) vii). In addition such further proposals shall be dealt with in accordance with Decisions 16.55 and 14.78 (Rev. CoP16).

On a proposal from the Secretariat, the Standing Committee can decide to cause this trade to cease partially or completely in the event of non-compliance by exporting or importing countries, or in the case of proven detrimental impacts of the trade on other elephant populations.

All other specimens shall be deemed to be specimens of species included in Appendix I and the trade in them shall be regulated accordingly.

[A1] Specimens of the domesticated form are not subject to the provisions of the Convention.

	Appendices I	II	III
ERETHIZONTIDAE New-world porcupines			
			Sphiggurus mexicanus (Honduras)<br>***Sphiggurus spinosus*** (Uruguay)
MURIDAE Mice, rats			
		Leporillus conditor<br>***Pseudomys fieldi***<br>***Xeromys myoides***<br>***Zyzomys pedunculatus***	
SCIURIDAE Ground squirrels, tree squirrels			
		Cynomys mexicanus<br>***Ratufa* spp.**	***Marmota caudata*** (India)<br>***Marmota himalayana*** (India)
SCANDENTIA Tree shrews			
		**SCANDENTIA spp.**	
SIRENIA			
DUGONGIDAE Dugong			
	Dugong dugon		
TRICHECHIDAE Manatees			
	Trichechus inunguis<br>***Trichechus manatus***<br>***Trichechus senegalensis***		

	Appendices I	II	III
**CLASS AVES (BIRDS)**			
ANSERIFORMES			
ANATIDAE Ducks, geese, swans, etc.			
	Anas aucklandica		
		Anas bernieri	
	Anas chlorotis		
		Anas formosa	
	Anas laysanensis		
	Anas nesiotis		
	Asarcornis scutulata		
		Branta canadensis leucopareia	
		Branta ruficollis	
	Branta sandvicensis		
		Coscoroba coscoroba	
		Cygnus melancoryphus	
		Dendrocygna arborea	
			Dendrocygna autumnalis (Honduras)
			Dendrocygna bicolor (Honduras)
		Oxyura leucocephala	
	Rhodonessa caryophyllacea		
		Sarkidiornis melanotos	
APODIFORMES			
TROCHILIDAE Hummingbirds			
		**TROCHILIDAE spp.** (Except the species included in Appendix I)	
	Glaucis dohrnii		
CHARADRIIFORMES			
BURHINIDAE Thick-knee			
			Burhinus bistriatus (Guatemala)
LARIDAE Relict gull			
	Larus relictus		

	Appendices I	II	III
SCOLOPACIDAE Curlews, greenshanks			
	Numenius borealis<br>***Numenius tenuirostris***<br>***Tringa guttifer***		
CICONIIFORMES			
BALAENICIPITIDAE Shoebill			
		Balaeniceps rex	
CICONIIDAE Storks			
	Ciconia boyciana<br><br>***Jabiru mycteria***<br>***Mycteria cinerea***	***Ciconia nigra***	
PHOENICOPTERIDAE Flamingos			
		**PHOENICOPTERIDAE spp.**	
THRESKIORNITHIDAE Ibises, spoonbills			
	Geronticus eremita<br>***Nipponia nippon***	***Eudocimus ruber***<br>***Geronticus calvus***<br><br>***Platalea leucorodia***	
COLUMBIFORMES			
COLUMBIDAE Doves, pigeons			
	Caloenas nicobarica<br>***Ducula mindorensis***	***Gallicolumba luzonica***<br>***Goura* spp.**	***Nesoenas mayeri*** (Mauritius)
CORACIIFORMES			
BUCEROTIDAE Hornbills			
	Aceros nipalensis	***Aceros* spp.** (Except the species included in Appendix I)<br><br>***Anorrhinus* spp.**<br>***Anthracoceros* spp.**	

	Appendices I	II	III
		***Berenicornis* spp.**	
		***Buceros* spp.** (Except the species included in Appendix I)	
	Buceros bicornis		
		***Penelopides* spp.**	
	Rhinoplax vigil		
		***Rhyticeros* spp.** (Except the species included in Appendix I)	
	Rhyticeros subruficollis		
CUCULIFORMES			
MUSOPHAGIDAE Turacos			
		***Tauraco* spp.**	
FALCONIFORMES Eagles, falcons, hawks, vultures			
		**FALCONIFORMES spp.** (Except the species included in Appendices I and III and *Caracara lutosa, Cathartes aura, Cathartes burrovianus, Cathartes melambrotus* and *Coragyps atratus* which are not included in the Appendices)	
ACCIPITRIDAE Hawks, eagles			
	Aquila adalberti		
	Aquila heliaca		
	Chondrohierax wilsonii		
	Haliaeetus albicilla		
	Harpia harpyja		
	Pithecophaga jefferyi		
CATHARTIDAE New-world vultures			
	Gymnogyps californianus		
			Sarcoramphus papa (Honduras)
	Vultur gryphus		
FALCONIDAE Falcons			
	Falco araeus		
	Falco jugger		

	Appendices I	II	III
	Falco newtoni (Only the population of Seychelles; all other populations are included in Appendix II)<br>***Falco peregrinus***<br>***Falco punctatus***<br>***Falco rusticolus***		
GALLIFORMES			
CRACIDAE Chachalacas, currassows, guans			
			Crax alberti (Colombia)
	Crax blumenbachii		
			Crax daubentoni (Colombia)<br>***Crax globulosa*** (Colombia)<br>***Crax rubra*** (Colombia, Guatemala, Honduras)
	Mitu mitu<br>***Oreophasis derbianus***		
			Ortalis vetula (Guatemala, Honduras)<br>***Pauxi pauxi*** (Colombia)
	Penelope albipennis		
			Penelope purpurascens (Honduras)<br>***Penelopina nigra*** (Guatemala)
	Pipile jacutinga<br>***Pipile pipile***		
MEGAPODIIDAE Maleo fowl			
	Macrocephalon maleo		
PHASIANIDAE Grouse, guineafowl, partridges, peafowl, pheasants, tragopans			
		Argusianus argus	
	Catreus wallichii<br>***Colinus virginianus ridgwayi***<br>***Crossoptilon crossoptilon***<br>***Crossoptilon mantchuricum***		
		Gallus sonneratii	

	Appendices I	II	III
		Ithaginis cruentus	
	Lophophorus impejanus		
	Lophophorus lhuysii		
	Lophophorus sclateri		
	Lophura edwardsi		
			Lophura leucomelanos (Pakistan)
	Lophura swinhoii		
			Meleagris ocellata (Guatemala)
			Pavo cristatus (Pakistan)
		Pavo muticus	
		Polyplectron bicalcaratum	
		Polyplectron germaini	
		Polyplectron malacense	
	Polyplectron napoleonis		
		Polyplectron schleiermacheri	
			Pucrasia macrolopha (Pakistan)
	Rheinardia ocellata		
	Syrmaticus ellioti		
	Syrmaticus humiae		
	Syrmaticus mikado		
		Syrmaticus reevesii	
	Tetraogallus caspius		
	Tetraogallus tibetanus		
	Tragopan blythii		
	Tragopan caboti		
	Tragopan melanocephalus		
			Tragopan satyra (Nepal)
		Tympanuchus cupido attwateri	
GRUIFORMES			
GRUIDAE Cranes			
		**GRUIDAE spp.** (Except the species included in Appendix **I**)	
	Antigone canadensis nesiotes		
	Antigone canadensis pulla		
	Antigone vipio		

	Appendices I	II	III
	Balearica pavonina<br>***Grus americana***<br>***Grus japonensis***<br>***Grus monacha***<br>***Grus nigricollis***<br>***Leucogeranus leucogeranus***		
OTIDIDAE Bustards			
	Ardeotis nigriceps<br>***Chlamydotis macqueenii***<br>***Chlamydotis undulata***<br>***Houbaropsis bengalensis***	**OTIDIDAE spp.** (Except the species included in Appendix I)	
RALLIDAE Lord Howe rail			
	Hypotaenidia sylvestris		
RHYNOCHETIDAE Kagu			
	Rhynochetos jubatus		
PASSERIFORMES			
ALAUDIDAE Larks			
			Alauda arvensis (Population of Ukraine) (Ukraine)<br>***Galerida cristata*** (Population of Ukraine) (Ukraine)<br>***Lullula arborea*** (Population of Ukraine) (Ukraine)<br>***Melanocorypha calandra*** (Population of Ukraine) (Ukraine)
ATRICHORNITHIDAE Western scrub-bird			
	Atrichornis clamosus		
COTINGIDAE Cotingas			
	Cotinga maculata<br>***Xipholena atropurpurea***	***Rupicola* spp.**	***Cephalopterus ornatus*** (Colombia)<br>***Cephalopterus penduliger*** (Colombia)

	Appendices I	II	III
EMBERIZIDAE Cardinals, tanagers			
			Emberiza citrinella (Population of Ukraine) (Ukraine)
			Emberiza hortulana (Population of Ukraine) (Ukraine)
		Gubernatrix cristata	
			Melopyrrha nigra (Cuba)
		Paroaria capitata	
		Paroaria coronata	
		Tangara fastuosa	
			Tiaris canorus (Cuba)
ESTRILDIDAE Mannikins, waxbills			
		Amandava formosa	
		Lonchura oryzivora	
		Poephila cincta cincta	
FRINGILLIDAE Finches			
			Carduelis cannabina (Population of Ukraine) (Ukraine)
			Carduelis carduelis (Population of Ukraine) (Ukraine)
	Carduelis cucullata		
			Carduelis flammea (Population of Ukraine) (Ukraine)
			Carduelis hornemanni (Population of Ukraine) (Ukraine)
			Carduelis spinus (Population of Ukraine) (Ukraine)
		Carduelis yarrellii	
			Carpodacus erythrinus (Population of Ukraine) (Ukraine)
			Loxia curvirostra (Population of Ukraine) (Ukraine)
			Pyrrhula pyrrhula (Population of Ukraine) (Ukraine)
			Serinus serinus (Population of Ukraine) (Ukraine)

	Appendices I	II	III
HIRUNDINIDAE White-eyed river martin			
	Pseudochelidon sirintarae		
ICTERIDAE Saffron-cowled blackbird			
	Xanthopsar flavus		
MELIPHAGIDAE Helmeted honeyeater			
		Lichenostomus melanops cassidix	
MUSCICAPIDAE Old-world flycatchers			
			Acrocephalus rodericanus (Mauritius)
		Copsychus malabaricus	
		Cyornis ruckii	
		Dasyornis broadbenti litoralis	
		Dasyornis longirostris	
			Erithacus rubecula (Population of Ukraine) (Ukraine)
			Ficedula parva (Population of Ukraine) (Ukraine)
		Garrulax canorus	
		Garrulax taewanus	
			Hippolais icterina (Population of Ukraine) (Ukraine)
		Leiothrix argentauris	
		Leiothrix lutea	
		Liocichla omeiensis	
			Luscinia luscinia (Population of Ukraine) (Ukraine)
			Luscinia megarhynchos (Population of Ukraine) (Ukraine)
			Luscinia svecica (Population of Ukraine) (Ukraine)
			Monticola saxatilis (Population of Ukraine) (Ukraine)
	Picathartes gymnocephalus		
	Picathartes oreas		
			Sylvia atricapilla (Population of Ukraine) (Ukraine)

	Appendices I	II	III
			Sylvia borin (Population of Ukraine) (Ukraine)<br>***Sylvia curruca*** (Population of Ukraine) (Ukraine)<br>***Sylvia nisoria*** (Population of Ukraine) (Ukraine)<br>***Terpsiphone bourbonnensis*** (Mauritius)<br>***Turdus merula*** (Population of Ukraine) (Ukraine)<br>***Turdus philomelos*** (Population of Ukraine) (Ukraine)
ORIOLIDAE Oriole			
			Oriolus oriolus (Population of Ukraine) (Ukraine)
PARADISAEIDAE Birds of paradise			
		**PARADISAEIDAE spp.**	
PARIDAE Tit			
			Parus ater (Population of Ukraine) (Ukraine)
PITTIDAE Pittas			
	Pitta gurneyi<br>***Pitta kochi***	***Pitta guajana***<br>***Pitta nympha***	
PYCNONOTIDAE Straw-headed bulbul			
	Pycnonotus zeylanicus		
STURNIDAE Mynas			
	Leucopsar rothschildi	***Gracula religiosa***	
TROGLODYTIDAE Wren			
			Troglodytes troglodytes (Population of Ukraine) (Ukraine)
ZOSTEROPIDAE White-chested white-eye			
	Zosterops albogularis		

	Appendices I	II	III
PELECANIFORMES			
FREGATIDAE Christmas frigatebird			
	Fregata andrewsi		
PELECANIDAE Dalmatian pelican			
	Pelecanus crispus		
SULIDAE Abbot's booby			
	Papasula abbotti		
PICIFORMES			
CAPITONIDAE Toucan barbet			
			Semnornis ramphastinus (Colombia)
PICIDAE Tristram's woodpecker			
	Dryocopus javensis richardsi		
RAMPHASTIDAE Toucans			
			Baillonius bailloni (Argentina)
		Pteroglossus aracari	
			Pteroglossus castanotis (Argentina)
		Pteroglossus viridis	
			Ramphastos dicolorus (Argentina)
		Ramphastos sulfuratus	
		Ramphastos toco	
		Ramphastos tucanus	
		Ramphastos vitellinus	
			Selenidera maculirostris (Argentina)
PODICIPEDIFORMES			
PODICIPEDIDAE Giant grebe			
	Podilymbus gigas		
PROCELLARIIFORMES			
DIOMEDEIDAE Short-tailed albatross			
		Phoebastria albatrus	

	Appendices I	II	III
PSITTACIFORMES			
		**PSITTACIFORMES spp.** (Except the species included in Appendix I and *Agapornis roseicollis, Melopsittacus undulatus, Nymphicus hollandicus* and *Psittacula krameri* which are not included in the Appendices)	
CACATUIDAE Cockatoos			
	Cacatua goffiniana<br>***Cacatua haematuropygia***<br>***Cacatua moluccensis***<br>***Cacatua sulphurea***<br>***Probosciger aterrimus***		
LORIIDAE Lories			
	Eos histrio<br>***Vini ultramarina***		
PSITTACIDAE Amazons, macaws, parakeets, parrots			
	Amazona arausiaca<br>***Amazona auropalliata***<br>***Amazona barbadensis***<br>***Amazona brasiliensis***<br>***Amazona finschi***<br>***Amazona guildingii***<br>***Amazona imperialis***<br>***Amazona leucocephala***<br>***Amazona oratrix***<br>***Amazona pretrei***<br>***Amazona rhodocorytha***<br>***Amazona tucumana***<br>***Amazona versicolor***<br>***Amazona vinacea***<br>***Amazona viridigenalis***<br>***Amazona vittata***<br>***Anodorhynchus* spp.**		

	Appendices I	II	III
	Ara ambiguus<br>***Ara glaucogularis***<br>***Ara macao***<br>***Ara militaris***<br>***Ara rubrogenys***<br>***Cyanopsitta spixii***<br>***Cyanoramphus cookii***<br>***Cyanoramphus forbesi***<br>***Cyanoramphus novaezelandiae***<br>***Cyanoramphus saisseti***<br>***Cyclopsitta diophthalma coxeni***<br>***Eunymphicus cornutus***<br>***Guarouba guarouba***<br>***Neophema chrysogaster***<br>***Ognorhynchus icterotis***<br>***Pezoporus flaviventris***<br>***Pezoporus occidentalis***<br>***Pezoporus wallicus***<br>***Pionopsitta pileata***<br>***Primolius couloni***<br>***Primolius maracana***<br>***Psephotellus chrysopterygius***<br>***Psephotellus dissimilis***<br>***Psephotellus pulcherrimus***<br>***Psittacula echo***<br>***Psittacus erithacus***<br>***Pyrrhura cruentata***<br>***Rhynchopsitta* spp.**<br>***Strigops habroptila***		
RHEIFORMES			
RHEIDAE Rheas			
	Pterocnemia pennata (Except the subspecies included in Appendix II)	***Pterocnemia pennata pennata***	

	Appendices I	II	III
		Rhea americana	
SPHENISCIFORMES			
SPHENISCIDAE Penguins			
	Spheniscus humboldti	***Spheniscus demersus***	
STRIGIFORMES Owls			
		**STRIGIFORMES spp.** (Except the species included in Appendix I and *Sceloglaux albifacies* which is not included in the Appendices)	
STRIGIDAE Owls			
	Heteroglaux blewitti<br>***Mimizuku gurneyi***<br>***Ninox natalis***		
TYTONIDAE Barn owls			
	Tyto soumagnei		
STRUTHIONIFORMES			
STRUTHIONIDAE Ostriches			
	Struthio camelus (Only the populations of Algeria, Burkina Faso, Cameroon, the Central African Republic, Chad, Mali, Mauritania, Morocco, the Niger, Nigeria, Senegal and the Sudan; no other population is included in the Appendices)		
TINAMIFORMES			
TINAMIDAE Solitary tinamou			
	Tinamus solitarius		
TROGONIFORMES			
TROGONIDAE Magnificent quetzal			
	Pharomachrus mocinno		

	Appendices I	II	III
**CLASS REPTILIA (REPTILES)**			
CROCODYLIA Alligators, caimans, crocodiles			
		**CROCODYLIA spp.** (Except the species included in Appendix I)	
ALLIGATORIDAE Alligators, caimans			
	Alligator sinensis<br>***Caiman crocodilus apaporiensis***<br>***Caiman latirostris*** (Except the populations of Argentina and Brazil[A11], which are included in Appendix II)<br>***Melanosuchus niger*** (Except the populations of Brazil and Ecuador [A12] which are included in Appendix II)		
CROCODYLIDAE Crocodiles			
	Crocodylus acutus (Except the populations of Colombia [Integrated Management District of Mangroves of the Bay of Cispata, Tinajones, La Balsa and Surrounding Areas, Department of Córdoba], Cuba and Mexico [A13] which are included in Appendix II)<br>***Crocodylus cataphractus***<br>***Crocodylus intermedius***<br>***Crocodylus mindorensis***<br>***Crocodylus moreletii*** (Except the populations of Belize [A14] and Mexico, which are included in Appendix II)		

[A11] The population of Brazil of *Caiman latirostris* is included in Appendix II subject to a zero annual export quota for wild specimens traded for commercial purposes.

[A12] The population of Ecuador of *Melanosuchus niger* is included in Appendix II subject to a zero annual export quota until an annual export quota has been approved by the CITES Secretariat and the IUCN/SSC Crocodile Specialist Group.

[A13] The population of Mexico of *Crocodylus acutus* is included in Appendix II subject to a zero export quota for wild specimens for commercial purposes.

[A14] The population of Belize of *Crocodylus moreletii* is included in Appendix II with a zero quota for wild specimens traded for commercial purposes.

	Appendices I	II	III
	Crocodylus niloticus (Except the populations of Botswana, Egypt[A15], Ethiopia, Kenya, Madagascar, Malawi, Mozambique, Namibia, South Africa, Uganda, the United Republic of Tanzania[A16], Zambia and Zimbabwe, which are included in Appendix **II**)<br>***Crocodylus palustris***<br>***Crocodylus porosus*** (Except the populations of Australia, Indonesia, Malaysia[A17], Papua New Guinea and the Philippines [population of the Palawan Islands][A18], which are included in Appendix **II**)<br>***Crocodylus rhombifer***<br>***Crocodylus siamensis***<br>***Osteolaemus tetraspis***<br>***Tomistoma schlegelii***		
GAVIALIDAE Indian gavial			
	Gavialis gangeticus		
RHYNCHOCEPHALIA			
SPHENODONTIDAE Tuatara			
	***Sphenodon* spp.**		
SAURIA			
AGAMIDAE Spiny-tailed lizards, agamas			
			Calotes ceylonensis (Sri Lanka)<br>***Calotes desilvai*** (Sri Lanka)<br>***Calotes liocephalus*** (Sri Lanka)

A15 The population of Egypt of *Crocodylus niloticus* is included in Appendix II subject to a zero quota for wild specimens traded for commercial purposes.

A16 The population of the United Republic of Tanzania of *Crocodylus niloticus* is included in Appendix II subject to an annual export quota of no more than 1,600 wild specimens including hunting trophies, in addition to ranched specimens.

A17 The population of Malaysia of *Crocodylus porosus* is included in Appendix II with wild harvest restricted to the State of Sarawak and a zero quota for wild specimens for the other States of Malaysia (Sabah and Peninsular Malaysia), with no change in the zero quota unless approved by the Parties.

A18 The population of the Palawan islands, Philippines, of *Crocodylus porosus* is included in Appendix II subject to a zero annual export quota for wild specimens traded for commercial purposes.

	Appendices I	II	III
			Calotes liolepis (Sri Lanka)
			Calotes manamendrai (Sri Lanka)
			Calotes nigrilabris (Sri Lanka)
			Calotes pethiyagodai (Sri Lanka)
		Ceratophora aspera A19	
	Ceratophora erdeleni		
	Ceratophora karu		
		Ceratophora stoddartii A19	
	Ceratophora tennentii		
	Cophotis ceylanica		
	Cophotis dumbara		
			***Ctenophorus* spp.** (Australia)
			***Intellagama* spp.** (Australia)
		Lyriocephalus scutatus A19	
		Physignathus cocincinus	
		***Saara* spp.**	
			***Tympanocryptis* spp.** (Australia)
		***Uromastyx* spp.**	
ANGUIDAE Alligator lizards			
		***Abronia* spp.** A20 (Except the species included in Appendix **I**)	
	Abronia anzuetoi		
	Abronia campbelli		
	Abronia fimbriata		
	Abronia frosti		
	Abronia meledona		
CHAMAELEONIDAE Chameleons			
		***Archaius* spp.**	
		***Bradypodion* spp.**	

A19 Zero export quota for wild specimens for commercial purposes.

A20 *Abronia aurita, A. gaiophantasma, A. montecristoi, A. salvadorensis* and *A. vasconcelosii* are subject to a zero export quota for wild specimens.

	Appendices I	II	III
	Brookesia perarmata	***Brookesia* spp.** (Except the species included in Appendix I)<br><br>***Calumma* spp.**<br>***Chamaeleo* spp.**<br>***Furcifer* spp.**<br>***Kinyongia* spp.**<br>***Nadzikambia* spp.**<br>***Palleon* spp.**<br>***Rhampholeon* spp.**<br>***Rieppeleon* spp.**<br>***Trioceros* spp.**	
CORDYLIDAE Spiny-tailed lizards			
		Cordylus* spp.**<br>Hemicordylus* spp.**<br>***Karusaurus* spp.**<br>***Namazonurus* spp.**<br>***Ninurta* spp.**<br>***Ouroborus* spp.**<br>***Pseudocordylus* spp.**<br>***Smaug* spp.**	
EUBLEPHARIDAE Eyelid geckos			
		***Goniurosaurus* spp.** (Except the species native to Japan)	***Goniurosaurus kuroiwae*** #18 (Japan)<br>***Goniurosaurus orientalis*** #18 (Japan)<br>***Goniurosaurus sengokui*** #18 (Japan)<br>***Goniurosaurus splendens*** #18 (Japan)<br>***Goniurosaurus toyamai*** #18 (Japan)<br>***Goniurosaurus yamashinae*** #18 (Japan)

#18 Excluding parts and derivatives, other than eggs.

	Appendices I	II	III
GEKKONIDAE Geckos			
			***Ailuronyx* spp.** (Seychelles)
			***Carphodactylus* spp.** (Australia)
	Cnemaspis psychedelica		
		Cyrtodactylus jeyporensis	
			***Dactylocnemis* spp.** (New Zealand)
		Gekko gecko	
	Gonatodes daudini		
			***Hoplodactylus* spp.** (New Zealand)
	Lygodactylus williamsi		
			***Mokopirirakau* spp.** (New Zealand)
		Nactus serpensinsula	
		***Naultinus* spp.**	
			***Nephrurus* spp.** (Australia)
			***Orraya* spp.** (Australia)
		Paroedura androyensis	
		Paroedura masobe	
		***Phelsuma* spp.**	
			***Phyllurus* spp.** (Australia)
		***Rhoptropella* spp.**	
			***Saltuarius* spp.** (Australia)
			Sphaerodactylus armasi (Cuba)
			Sphaerodactylus celicara (Cuba)
			Sphaerodactylus dimorphicus (Cuba)
			Sphaerodactylus intermedius (Cuba)
			Sphaerodactylus nigropunctatus alayoi (Cuba)
			Sphaerodactylus nigropunctatus granti (Cuba)
			Sphaerodactylus nigropunctatus lissodesmus (Cuba)
			Sphaerodactylus nigropunctatus ocujal (Cuba)
			Sphaerodactylus nigropunctatus strategus (Cuba)

	Appendices I	II	III
			Sphaerodactylus notatus atactus (Cuba)
			Sphaerodactylus oliveri (Cuba)
			Sphaerodactylus pimienta (Cuba)
			Sphaerodactylus ruibali (Cuba)
			Sphaerodactylus siboney (Cuba)
			Sphaerodactylus torrei (Cuba)
			***Strophurus* spp.** (Australia)
		Tarentola chazaliae	
			***Toropuku* spp.** (New Zealand)
			***Tukutuku* spp.** (New Zealand)
			***Underwoodisaurus* spp.** (Australia)
		***Uroplatus* spp.**	
			***Uvidicolus* spp.** (Australia)
			***Woodworthia* spp.** (New Zealand)
HELODERMATIDAE Beaded lizards, Gila monster			
	Heloderma horridum charlesbogerti	***Heloderma* spp.** (Except the subspecies included in Appendix I)	
IGUANIDAE Iguanas			
		Amblyrhynchus cristatus	
	***Brachylophus* spp.**		
		***Conolophus* spp.**	
		***Ctenosaura* spp.**	
	***Cyclura* spp.**		
		***Iguana* spp.**	
	Sauromalus varius		
LACERTIDAE Lizards			
	Gallotia simonyi		
		Podarcis lilfordi	
		Podarcis pityusensis	

	Appendices I	II	III
LANTHANOTIDAE Earless monitor lizards			
		**LANTHANOTIDAE spp.** [A19]	
PHRYNOSOMATIDAE Horned lizards			
		***Phrynosoma* spp.**	
POLYCHROTIDAE Anoles			
			Anolis agueroi (Cuba)<br>***Anolis baracoae*** (Cuba)<br>***Anolis barbatus*** (Cuba)<br>***Anolis chamaeleonides*** (Cuba)<br>***Anolis equestris*** (Cuba)<br>***Anolis guamuhaya*** (Cuba)<br>***Anolis luteogularis*** (Cuba)<br>***Anolis pigmaequestris*** (Cuba)<br>***Anolis porcus*** (Cuba)
SCINCIDAE Solomon Islands giant skink			
	Tiliqua adelaidensis	***Corucia zebrata***	***Egernia* spp.** (Australia)<br>***Tiliqua multifasciata*** (Australia)<br>***Tiliqua nigrolutea*** (Australia)<br>***Tiliqua occipitalis*** (Australia)<br>***Tiliqua rugosa*** (Australia)<br>***Tiliqua scincoides intermedia*** (Australia)<br>***Tiliqua scincoides scincoides*** (Australia)
TEIIDAE Caiman lizards, tegu lizards			
		Crocodilurus amazonicus<br>***Dracaena* spp.**<br>***Salvator* spp.**	

[A19] Zero export quota for wild specimens for commercial purposes.

	Appendices I	II	III
		***Tupinambis* spp.**	
VARANIDAE Monitor lizards			
	Varanus bengalensis<br>***Varanus flavescens***<br>***Varanus griseus***<br>***Varanus komodoensis***<br>***Varanus nebulosus***	***Varanus* spp.** (Except the species included in Appendix I)	
XENOSAURIDAE Chinese crocodile lizard			
	Shinisaurus crocodilurus		
SERPENTES			
BOIDAE Boas			
	Acrantophis* spp.**<br>Boa constrictor occidentalis***<br>***Chilabothrus monensis***<br>***Chilabothrus subflavus***<br>***Sanzinia madagascariensis***	**BOIDAE spp.** (Except the species included in Appendix I)	
BOLYERIIDAE Round Island boas			
	Bolyeria multocarinata<br>***Casarea dussumieri***	**BOLYERIIDAE spp.** (Except the species included in Appendix I)	
COLUBRIDAE Typical snakes, water snakes, whipsnakes			
		Clelia clelia<br>***Cyclagras gigas***<br>***Elachistodon westermanni***<br>***Ptyas mucosus***	***Atretium schistosum*** (India)<br>***Cerberus rynchops*** (India)<br>***Xenochrophis piscator*** (India)

	Appendices I	II	III
			Xenochrophis schnurrenbergeri (India)<br>***Xenochrophis tytleri*** (India)
ELAPIDAE Cobras, coral snakes			
		Hoplocephalus bungaroides	
			Micrurus diastema (Honduras)<br>***Micrurus nigrocinctus*** (Honduras)<br>***Micrurus ruatanus*** (Honduras)
		Naja atra<br>***Naja kaouthia***<br>***Naja mandalayensis***<br>***Naja naja***<br>***Naja oxiana***<br>***Naja philippinensis***<br>***Naja sagittifera***<br>***Naja samarensis***<br>***Naja siamensis***<br>***Naja sputatrix***<br>***Naja sumatrana***<br>***Ophiophagus hannah***	
LOXOCEMIDAE Mexican dwarf boas			
		**LOXOCEMIDAE spp.**	
PYTHONIDAE Pythons			
		**PYTHONIDAE spp.** (Except the species included in Appendix I)	
	Python molurus		
TROPIDOPHIIDAE Wood boas			
		**TROPIDOPHIIDAE spp.**	
VIPERIDAE Vipers			
		Atheris desaixi<br>***Bitis worthingtoni***	
			Crotalus durissus (Honduras)<br>***Daboia palaestinae*** (Israel)<br>***Daboia russelii*** (India)

	Appendices I	II	III
	Vipera ursinii (Only the population of Europe, except the area which formerly constituted the Union of Soviet Socialist Republics; these latter populations are not included in the Appendices)	***Montivipera wagneri***<br>***Protobothrops mangshanensis***<br>***Pseudocerastes urarachnoides***	
TESTUDINES			
CARETTOCHELYIDAE Pig-nosed turtle			
		Carettochelys insculpta	
CHELIDAE Roti snake-necked turtle, Western swamp tortoise			
	Pseudemydura umbrina	***Chelodina mccordi*** [A21]<br>***Chelus fimbriatus*** {Includes ***Chelus orinocensis***}	
CHELONIIDAE Sea turtles			
	**CHELONIIDAE spp.**		
CHELYDRIDAE Snapping turtles			
		Chelydra serpentina<br>***Macrochelys temminckii***	
DERMATEMYDIDAE Central American river turtles			
		Dermatemys mawii	
DERMOCHELYIDAE Leatherback turtles			
	Dermochelys coriacea		
EMYDIDAE Box turtles, freshwater turtles			
		Clemmys guttata<br>***Emydoidea blandingii***	***Emys orbicularis*** (Population of Ukraine) (Ukraine)

[A21] Zero export quota for specimens from the wild.

	Appendices I	II	III
		Glyptemys insculpta	
	Glyptemys muhlenbergii		
			***Graptemys* spp.** (Except the species included in Appendix **II**) (United States of America)
		Graptemys barbouri	
		Graptemys ernsti	
		Graptemys gibbonsi	
		Graptemys pearlensis	
		Graptemys pulchra	
		Malaclemys terrapin	
		***Terrapene* spp.** (Except the species included in Appendix **I**)	
	Terrapene coahuila		
GEOEMYDIDAE Box turtles, freshwater turtles			
	Batagur affinis		
	Batagur baska		
		Batagur borneoensis [A22]	
		Batagur dhongoka	
	Batagur kachuga		
		Batagur trivittata [A22]	
		***Cuora* spp.** [A22] (Except the species included in Appendix **I**)	
	Cuora bourreti		
	Cuora galbinifrons		
	Cuora picturata		
		***Cyclemys* spp.**	
	Geoclemys hamiltonii		
		Geoemyda japonica	
		Geoemyda spengleri	
		Hardella thurjii	

[A22] Zero quota for wild specimens for commercial purposes.

[A22] For *Cuora aurocapitata, C. flavomarginata, C. mccordi, C. mouhotii, C. pani, C. trifasciata, C. yunnanensis* and *C. zhoui*: a zero quota for wild specimens for commercial purposes.

	Appendices<br>I	 II	 III
		Heosemys annandalii [A22]	
		Heosemys depressa [A22]	
		Heosemys grandis	
		Heosemys spinosa	
		Leucocephalon yuwonoi	
		Malayemys khoratensis	
		Malayemys macrocephala	
		Malayemys subtrijuga	
	Mauremys annamensis		
		Mauremys japonica	
		Mauremys mutica	
		Mauremys nigricans	
			Mauremys reevesii (China)
			Mauremys sinensis (China)
	Melanochelys tricarinata		
		Melanochelys trijuga	
	Morenia ocellata		
		Morenia petersi	
		Notochelys platynota	
		Orlitia borneensis [A22]	
		***Pangshura* spp.** (Except the species included in Appendix I)	
	Pangshura tecta		
		***Rhinoclemmys* spp.**	
		Sacalia bealei	
		Sacalia quadriocellata	
		Siebenrockiella crassicollis	
		Siebenrockiella leytensis	
		Vijayachelys silvatica	
KINOSTERNIDAE Mud turtles			
		Claudius angustatus	

[A22] Zero quota for wild specimens for commercial purposes.

	Appendices<br>I	 II	 III
	 <br>***Kinosternon cora***<br>***Kinosternon vogti***	***Kinosternon* spp.** (Except the species included in Appendix **I**)<br><br><br>***Staurotypus salvinii***<br>***Staurotypus triporcatus***<br>***Sternotherus* spp.**	
PLATYSTERNIDAE Big-headed turtles			
	**PLATYSTERNIDAE spp.**		
PODOCNEMIDIDAE Afro-American sideneck turtles			
		Erymnochelys madagascariensis<br>***Peltocephalus dumerilianus***<br>***Podocnemis* spp.**	
TESTUDINIDAE Tortoises			
	 <br>***Astrochelys radiata***<br>***Astrochelys yniphora***<br>***Chelonoidis niger***<br>***Geochelone elegans***<br>***Geochelone platynota***<br>***Gopherus flavomarginatus***<br>***Malacochersus tornieri***<br>***Psammobates geometricus***<br>***Pyxis arachnoides***<br>***Pyxis planicauda***<br>***Testudo kleinmanni***	**TESTUDINIDAE spp.** [A23] (Except the species included in Appendix **I**)	
TRIONYCHIDAE Softshell turtles			
		Amyda cartilaginea<br>***Apalone* spp.** (Except the subspecies included in Appendix **I**)	

[A23] For *Centrochelys sulcata:* a zero annual export quota has been established for specimens removed from the wild and traded for primarily commercial purposes.

	Appendices<br>I	 II	 III
	Apalone spinifera atra		
		***Chitra* spp.** (Except the species included in Appendix I)	
	Chitra chitra		
	Chitra vandijki		
		Cyclanorbis elegans	
		Cyclanorbis senegalensis	
		Cycloderma aubryi	
		Cycloderma frenatum	
		Dogania subplana	
		Lissemys ceylonensis	
		Lissemys punctata	
		Lissemys scutata	
		Nilssonia formosa	
	Nilssonia gangetica		
	Nilssonia hurum		
	Nilssonia leithii		
	Nilssonia nigricans		
		Palea steindachneri	
		***Pelochelys* spp.**	
		Pelodiscus axenaria	
		Pelodiscus maackii	
		Pelodiscus parviformis	
		Rafetus euphraticus	
		Rafetus swinhoei	
		Trionyx triunguis	

	Appendices I	II	III
**CLASS AMPHIBIA (AMPHIBIANS)**			
ANURA			
AROMOBATIDAE Cryptic forest frogs			
		Allobates femoralis<br>***Allobates hodli***<br>***Allobates myersi***<br>***Allobates zaparo***<br>***Anomaloglossus rufulus***	
BUFONIDAE Toads			
	Altiphrynoides* spp.**<br>Atelopus zeteki***<br>***Incilius periglenes***<br>***Nectophrynoides* spp.**<br>***Nimbaphrynoides* spp.**<br>***Sclerophrys channingi***<br>***Sclerophrys superciliaris***		
CALYPTOCEPHALELLIDAE Chilean toad			
			Calyptocephalella gayi (Chile)
CENTROLENIDAE Glass frogs			
		**CENTROLENIDAE spp.**	
DENDROBATIDAE Poison frogs			
		Adelphobates* spp.**<br>Ameerega* spp.**<br>***Andinobates* spp.**<br>***Dendrobates* spp.**<br>***Epipedobates* spp.**<br>***Excidobates* spp.**<br>***Hyloxalus azureiventris***<br>***Minyobates* spp.**<br>***Oophaga* spp.**<br>***Paruwrobates andinus***<br>***Paruwrobates erythromos***<br>***Phyllobates* spp.**<br>***Ranitomeya* spp.**	

	Appendices I	II	III
DICROGLOSSIDAE Indian bullfrogs			
		Euphlyctis hexadactylus<br>***Hoplobatrachus tigerinus***	
HYLIDAE Tree frogs			
		***Agalychnis* spp.**[A24] {Includes *Agalychnis annae, A. callidryas, A. lemur, A. moreletii, A. saltator, A. spurrelli* and *A. terranova*}	
MANTELLIDAE Mantella frogs			
		***Mantella* spp.**	
MICROHYLIDAE Tomato frogs			
		Dyscophus antongilii<br>***Dyscophus guineti***<br>***Dyscophus insularis***<br>***Scaphiophryne boribory***<br>***Scaphiophryne gottlebei***<br>***Scaphiophryne marmorata***<br>***Scaphiophryne spinosa***	
MYOBATRACHIDAE Gastric-brooding frogs			
		***Rheobatrachus* spp.** (Except *Rheobatrachus silus* and *Rheobatrachus vitellinus* which are not included in the Appendices)	
TELMATOBIIDAE Titicaca water frog			
	Telmatobius culeus		
CAUDATA			
AMBYSTOMATIDAE Axolotl, mole salamander			
		Ambystoma dumerilii<br>***Ambystoma mexicanum***	

[A24] For *Agalychnis lemur*: a zero annual export quota for wild-taken specimens traded for commercial purposes.

	Appendices I	II	III
CRYPTOBRANCHIDAE Giant salamanders			
	***Andrias* spp.**		***Cryptobranchus alleganiensis*** (United States of America)
HYNOBIIDAE Asiatic salamander			
			Hynobius amjiensis (China)
SALAMANDRIDAE Newts and salamanders			
	Neurergus kaiseri	***Echinotriton chinhaiensis***<br>***Echinotriton maxiquadratus***<br>***Laotriton laoensis***[A24]<br>***Paramesotriton* spp.**<br>***Tylototriton* spp.**	***Echinotriton andersoni*** [#18] (Japan)<br>***Salamandra algira*** (Algeria)
**CLASS ELASMOBRANCHII (SHARKS)**			
CARCHARHINIFORMES			
CARCHARHINIDAE Requiem sharks			
		**CARCHARHINIDAE spp.**	
SPHYRNIDAE Hammerhead sharks			
		**SPHYRNIDAE spp.**	
LAMNIFORMES			
ALOPIIDAE Thresher sharks			
		***Alopias* spp.**	
CETORHINIDAE Basking shark			
		Cetorhinus maximus	
LAMNIDAE Mackerel sharks			
		Carcharodon carcharias	

[#18] Excluding parts and derivatives, other than eggs.

[A24] A zero annual export quota for wild-taken specimens traded for commercial purposes.

	Appendices I	II	III
		Isurus oxyrinchus<br>***Isurus paucus***<br>***Lamna nasus***	
MYLIOBATIFORMES			
MYLIOBATIDAE Eagle and mobulid rays			
		***Mobula* spp.**	
POTAMOTRYGONIDAE Freshwater stingrays			
			Paratrygon aiereba (Colombia)
			***Potamotrygon* spp.** (Only the populations of Brazil not included in Appendix II) (Brazil)
		Potamotrygon albimaculata	
			Potamotrygon constellata (Colombia)
		Potamotrygon henlei	
		Potamotrygon jabuti	
		Potamotrygon leopoldi	
			Potamotrygon magdalenae (Colombia)
		Potamotrygon marquesi	
			Potamotrygon motoro (Colombia)
			Potamotrygon orbignyi (Colombia)
			Potamotrygon schroederi (Colombia)
			Potamotrygon scobina (Colombia)
		Potamotrygon signata	
		Potamotrygon wallacei	
			Potamotrygon yepezi (Colombia)
ORECTOLOBIFORMES			
RHINCODONTIDAE Whale shark			
		Rhincodon typus	
PRISTIFORMES			
PRISTIDAE Sawfishes			
	**PRISTIDAE spp.**		

	Appendices I	II	III
RHINOPRISTIFORMES			
GLAUCOSTEGIDAE Guitarfishes			
		***Glaucostegus* spp.**	
RHINIDAE Wedgefishes			
		**RHINIDAE spp.**	
RHINOBATIDAE Guitarfishes			
		**RHINOBATIDAE spp.**	
**CLASS ACTINOPTERI (FISHES)**			
ACIPENSERIFORMES			
		**ACIPENSERIFORMES spp.** (Except the species included in Appendix I)	
ACIPENSERIDAE Sturgeons			
	Acipenser brevirostrum<br>***Acipenser sturio***		
ANGUILLIFORMES			
ANGUILLIDAE River eel			
		Anguilla anguilla	
CYPRINIFORMES			
CATOSTOMIDAE Cui-ui			
	Chasmistes cujus		
CYPRINIDAE Carps			
	Probarbus jullieni	***Caecobarbus geertsii***	
OSTEOGLOSSIFORMES			
ARAPAIMIDAE Arapaima			
		Arapaima gigas	
OSTEOGLOSSIDAE Bonytongues			
	Scleropages formosus<br>***Scleropages inscriptus***		

	Appendices I	II	III
PERCIFORMES			
LABRIDAE Humphead wrasse			
		Cheilinus undulatus	
POMACANTHIDAE Angelfish			
		Holacanthus clarionensis	***Holacanthus limbaughi*** (France)
SCIAENIDAE Totoaba			
	Totoaba macdonaldi		
SILURIFORMES			
LORICARIIDAE Armoured catfish			
		Hypancistrus zebra [A19]	
PANGASIIDAE Giant catfish			
	Pangasianodon gigas		
SYNGNATHIFORMES			
SYNGNATHIDAE Seahorses			
		***Hippocampus* spp.**	
**CLASS DIPNEUSTI (LUNGFISHES)**			
CERATODONTIFORMES			
NEOCERATODONTIDAE Queensland lungfish			
		Neoceratodus forsteri	
**CLASS COELACANTHI (COELACANTHS)**			
COELACANTHIFORMES			
LATIMERIIDAE Coelacanths			
	***Latimeria* spp.**		

[A19] A zero export quota for wild specimens for commercial purposes.

	Appendices I	Appendices II	Appendices III
**PHYLUM ECHINODERMATA**<br>**CLASS HOLOTHUROIDEA (SEA CUCUMBERS)**			
ASPIDOCHIROTIDA			
STICHOPODIDAE Sea cucumbers			
		***Thelenota* spp.**	***Isostichopus fuscus*** (Ecuador)
HOLOTHURIIDA			
HOLOTHURIIDAE Teatfishes, sea cucumbers			
		Holothuria fuscogilva<br>***Holothuria nobilis***<br>***Holothuria whitmaei***	
**PHYLUM ARTHROPODA**<br>**CLASS ARACHNIDA (SCORPIONS AND SPIDERS)**			
ARANEAE			
THERAPHOSIDAE Red-kneed tarantulas, tarantulas			
		Aphonopelma pallidum<br>***Brachypelma* spp.**<br>***Poecilotheria* spp.**<br>***Sericopelma angustum***<br>***Sericopelma embrithes***<br>***Tliltocatl* spp.**	***Caribena versicolor*** (European Union)
SCORPIONES			
SCORPIONIDAE Scorpions			
		Pandinus camerounensis<br>***Pandinus dictator***<br>***Pandinus gambiensis***<br>***Pandinus imperator***<br>***Pandinus roeseli***	

	Appendices I	II	III
**CLASS INSECTA (INSECTS)**			
COLEOPTERA			
LUCANIDAE Stag beetles			
			***Colophon* spp.** (South Africa)
SCARABAEIDAE Satanas beetle			
		Dynastes satanas	
LEPIDOPTERA			
NYMPHALIDAE Brush-footed butterflies			
			Agrias amydon boliviensis (Plurinational State of Bolivia)<br>***Morpho godartii lachaumei*** (Plurinational State of Bolivia)<br>***Prepona praeneste buckleyana*** (Plurinational State of Bolivia)
PAPILIONIDAE Birdwing butterflies, swallowtail butterflies			
	Achillides chikae chikae<br>***Achillides chikae hermeli***		
		Atrophaneura jophon<br>***Atrophaneura pandiyana***<br>***Bhutanitis* spp.**<br>***Ornithoptera* spp.** (Except the species included in Appendix I)	
	Ornithoptera alexandrae<br>***Papilio homerus***		
		Papilio hospiton	
			Papilio phorbanta (European Union)
	Parides burchellanus		
		Parnassius apollo<br>***Teinopalpus* spp.**<br>***Trogonoptera* spp.**<br>***Troides* spp.**	

	Appendices I	II	III
**PHYLUM ANNELIDA**<br>**CLASS HIRUDINOIDEA (LEECHES)**			
ARHYNCHOBDELLIDA			
HIRUDINIDAE Medicinal leeches			
		Hirudo medicinalis<br>***Hirudo verbana***	
**PHYLUM MOLLUSCA**<br>**CLASS BIVALVIA (CLAMS AND MUSSELS)**			
MYTILOIDA			
MYTILIDAE Date mussel			
		Lithophaga lithophaga	
UNIONOIDA			
UNIONIDAE Freshwater mussels, pearly mussels			
	Conradilla caelata		
		Cyprogenia aberti	
	Dromus dromas		
	Epioblasma curtisi		
	Epioblasma florentina		
	Epioblasma sampsonii		
	Epioblasma sulcata perobliqua		
	Epioblasma torulosa gubernaculum		
		Epioblasma torulosa rangiana	
	Epioblasma torulosa torulosa		
	Epioblasma turgidula		
	Epioblasma walkeri		
	Fusconaia cuneolus		
	Fusconaia edgariana		
	Lampsilis higginsii		
	Lampsilis orbiculata orbiculata		
	Lampsilis satur		
	Lampsilis virescens		
	Plethobasus cicatricosus		
	Plethobasus cooperianus		

	Appendices<br>I	 II	 III
	Pleurobema plenum<br>*Potamilus capax*<br>*Quadrula intermedia*<br>*Quadrula sparsa*<br>*Toxolasma cylindrella*<br>*Unio nickliniana*<br>*Unio tampicoensis tecomatensis*<br>*Villosa trabalis*	***Pleurobema clava***	
VENEROIDA			
TRIDACNIDAE Giant clams			
		**TRIDACNIDAE spp.**	
**CLASS CEPHALOPODA (SQUIDS, OCTOPUSES, CUTTLEFISH)**			
NAUTILIDA			
NAUTILIDAE Chambered nautilus			
		**NAUTILIDAE spp.**	
**CLASS GASTROPODA (SNAILS AND CONCHES)**			
MESOGASTROPODA			
STROMBIDAE Queen conch			
		Strombus gigas	
STYLOMMATOPHORA			
ACHATINELLIDAE Agate snails, oahu tree snails			
	***Achatinella* spp.**		
CAMAENIDAE Green tree snail			
		Papustyla pulcherrima	
CEPOLIDAE Helicoid terrestrial snails			
	***Polymita* spp.**		
**PHYLUM CNIDARIA**<br>**CLASS ANTHOZOA (CORALS AND SEA ANEMONES)**			
ANTIPATHARIA Black corals			
		**ANTIPATHARIA spp.**	

	Appendices I	II	III
GORGONACEAE			
CORALLIIDAE Red and pink corals			
			Corallium elatius (China)<br>***Corallium japonicum*** (China)<br>***Corallium konjoi*** (China)<br>***Corallium secundum*** (China)
HELIOPORACEA			
HELIOPORIDAE Blue corals			
		**HELIOPORIDAE spp.** [A25] {Includes only the species *Heliopora coerulea*}	
SCLERACTINIA Stony corals			
		**SCLERACTINIA spp.** [A25]	
STOLONIFERA			
TUBIPORIDAE Organ-pipe corals			
		**TUBIPORIDAE spp.**[A25]	
**CLASS HYDROZOA (SEA FERNS, FIRE CORALS AND STINGING MEDUSAE)**			
MILLEPORINA			
MILLEPORIDAE Fire corals			
		**MILLEPORIDAE spp.** [A25]	
STYLASTERINA			
STYLASTERIDAE Lace corals			
		**STYLASTERIDAE spp.** [A25]	

[A25] Fossils are not subject to the provisions of the Convention.

	Appendices I	II	III
**FLORA (PLANTS)**			
AGAVACEAE Agaves, bear-grass			
	Agave parviflora	***Agave victoriae-reginae*** #4<br>***Nolina interrata***<br>***Yucca queretaroensis***	
AIZOACEAE Stone plants			
			Conophytum* spp.** (South Africa)<br>Mestoklema tuberosum*** (South Africa)
AMARYLLIDACEAE Snowdrops, sternbergias			
		Galanthus* spp.** #4<br>Sternbergia* spp.** #4	
ANACARDIACEAE Cashews			
		Operculicarya decaryi<br>***Operculicarya hyphaenoides***<br>***Operculicarya pachypus***	
APOCYNACEAE Elephant trunks, hoodias, snakewood			
		***Hoodia* spp.** #9	

#4 All parts and derivatives, except:

a) seeds (including seedpods of Orchidaceae), spores and pollen (including pollinia). The exemption does not apply to seeds from Cactaceae spp. exported from Mexico, and to seeds from *Beccariophoenix madagascariensis* and *Dypsis decaryi* exported from Madagascar;
b) seedling or tissue cultures obtained *in vitro* transported in sterile containers;
c) cut flowers of artificially propagated plants;
d) fruits, and parts and derivatives thereof, of naturalized or artificially propagated plants of the genus *Vanilla* (Orchidaceae) and of the family Cactaceae;
e) stems, flowers, and parts and derivatives thereof, of naturalized or artificially propagated plants of the genera *Opuntia* subgenus *Opuntia* and *Selenicereus* (Cactaceae);
f) finished products of *Aloe ferox* and *Euphorbia antisyphilitica* packaged and ready for retail trade; and
g) finished products derived from artificial propagation, packaged and ready for retail trade of cosmetics containing parts and derivatives of *Bletilla striata, Cycnoches cooperi, Gastrodia elata, Phalaenopsis amabilis* or *Phalaenopsis lobbii*.

#9 All parts and derivatives except those bearing a label:
"Produced from *Hoodia* spp. material obtained through controlled harvesting and production under the terms of an agreement with the relevant CITES Management Authority of [Botswana under agreement No. BW/xxxxxx] [Namibia under agreement No. NA/xxxxxx] [South Africa under agreement No. ZA/xxxxxx]".

	Appendices I	II	III
		***Pachypodium* spp.** #4 (Except the species included in Appendix I)	
	Pachypodium ambongense ***Pachypodium baronii*** ***Pachypodium decaryi*** ***Pachypodium windsorii***		
			Raphionacme zeyheri (South Africa)
		Rauvolfia serpentina #2	
ARALIACEAE Ginseng			
		Panax ginseng #3 (Only the population of the Russian Federation; no other population is included in the Appendices) ***Panax quinquefolius*** #3	
ARAUCARIACEAE Monkey puzzle			
	Araucaria araucana		
ASPARAGACEAE Beaucarnea			
		***Beaucarnea* spp.**	
BERBERIDACEAE Himalyan may-apple			
		Podophyllum hexandrum #2	
BIGNONIACEAE Trumpet trees			
		***Handroanthus* spp.**#17	

#4 All parts and derivatives, except:

a) seeds (including seedpods of Orchidaceae), spores and pollen (including pollinia). The exemption does not apply to seeds from Cactaceae spp. exported from Mexico, and to seeds from *Beccariophoenix madagascariensis* and *Dypsis decaryi* exported from Madagascar;
b) seedling or tissue cultures obtained *in vitro* transported in sterile containers;
c) cut flowers of artificially propagated plants;
d) fruits, and parts and derivatives thereof, of naturalized or artificially propagated plants of the genus *Vanilla* (Orchidaceae) and of the family Cactaceae;
e) stems, flowers, and parts and derivatives thereof, of naturalized or artificially propagated plants of the genera *Opuntia* subgenus *Opuntia* and *Selenicereus* (Cactaceae);
f) finished products of *Aloe ferox* and *Euphorbia antisyphilitica* packaged and ready for retail trade; and
g) finished products derived from artificial propagation, packaged and ready for retail trade of cosmetics containing parts and derivatives of *Bletilla striata, Cycnoches cooperi, Gastrodia elata, Phalaenopsis amabilis* or *Phalaenopsis lobbii*.

#2 All parts and derivatives except:

a) seeds and pollen; and
b) finished products packaged and ready for retail trade.

#3 Whole and sliced roots and parts of roots, excluding manufactured parts or derivatives, such as powders, pills, extracts, tonics, teas and confectionery.

#17 Logs, sawn wood, veneer sheets, plywood and transformed wood.

	Appendices<br>I	II	III
		Roseodendron* spp.**[#17]<br>Tabebuia* spp.**[#17]	
BROMELIACEAE Tillandsias			
		Tillandsia harrisii [#4]<br>***Tillandsia kammii*** [#4]<br>***Tillandsia xerographica*** [#4]	
CACTACEAE Cacti			
	***Ariocarpus* spp.**	**CACTACEAE spp.**[P1] [#4] (Except the species included in Appendix I and *Pereskia* spp., *Pereskiopsis* spp. and *Quiabentia* spp. which are not included in the Appendices)	

[#4] All parts and derivatives, except:

a) seeds (including seedpods of Orchidaceae), spores and pollen (including pollinia). The exemption does not apply to seeds from Cactaceae spp. exported from Mexico, and to seeds from *Beccariophoenix madagascariensis* and *Dypsis decaryi* exported from Madagascar;
b) seedling or tissue cultures obtained *in vitro* transported in sterile containers;
c) cut flowers of artificially propagated plants;
d) fruits, and parts and derivatives thereof, of naturalized or artificially propagated plants of the genus *Vanilla* (Orchidaceae) and of the family Cactaceae;
e) stems, flowers, and parts and derivatives thereof, of naturalized or artificially propagated plants of the genera *Opuntia* subgenus *Opuntia* and *Selenicereus* (Cactaceae);
f) finished products of *Aloe ferox* and *Euphorbia antisyphilitica* packaged and ready for retail trade; and
g) finished products derived from artificial propagation, packaged and ready for retail trade of cosmetics containing parts and derivatives of *Bletilla striata, Cycnoches cooperi, Gastrodia elata, Phalaenopsis amabilis* or *Phalaenopsis lobbii*..

[P1] Artificially propagated specimens of the following hybrids and/or cultivars are not subject to the provisions of the Convention:

– *Hatiora* x *graeseri*
– *Schlumbergera* x *buckleyi*
– *Schlumbergera russelliana* x *Schlumbergera truncata*
– *Schlumbergera orssichiana* x *Schlumbergera truncata*
– *Schlumbergera opuntioides* x *Schlumbergera truncata*
– *Schlumbergera truncata* (cultivars)
– Cactaceae spp. colour mutants grafted on the following grafting stocks: *Harrisia* 'Jusbertii', *Hylocereus trigonus* or *Hylocereus undatus*
– *Opuntia microdasys* (cultivars).

[#4] All parts and derivatives, except:

a) seeds (including seedpods of Orchidaceae), spores and pollen (including pollinia). The exemption does not apply to seeds from Cactaceae spp. exported from Mexico, and to seeds from *Beccariophoenix madagascariensis* and *Dypsis decaryi* exported from Madagascar;
b) seedling or tissue cultures obtained *in vitro* transported in sterile containers;
c) cut flowers of artificially propagated plants;
d) fruits, and parts and derivatives thereof, of naturalized or artificially propagated plants of the genus *Vanilla* (Orchidaceae) and of the family Cactaceae;
e) stems, flowers, and parts and derivatives thereof, of naturalized or artificially propagated plants of the genera *Opuntia* subgenus *Opuntia* and *Selenicereus* (Cactaceae);
f) finished products of *Aloe ferox* and *Euphorbia antisyphilitica* packaged and ready for retail trade; and
g) finished products derived from artificial propagation, packaged and ready for retail trade of cosmetics containing parts and derivatives of *Bletilla striata, Cycnoches cooperi, Gastrodia elata, Phalaenopsis amabilis* or *Phalaenopsis lobbii*..

	Appendices I	II	III
	Astrophytum asterias		
	Aztekium ritteri		
	Coryphantha werdermannii		
	***Discocactus* spp.**		
	Echinocereus ferreiranus* ssp. *lindsayorum		
	Echinocereus schmollii		
	Escobaria minima		
	Escobaria sneedii		
	Mammillaria pectinifera {Includes **ssp. *solisioides***}		
	Melocactus conoideus		
	Melocactus deinacanthus		
	Melocactus glaucescens		
	Melocactus paucispinus		
	Obregonia denegrii		
	Pachycereus militaris		
	Pediocactus bradyi		
	Pediocactus knowltonii		
	Pediocactus paradinei		
	Pediocactus peeblesianus		
	Pediocactus sileri		
	***Pelecyphora* spp.**		
	Sclerocactus blainei		
	Sclerocactus brevihamatus* ssp. *tobuschii		
	Sclerocactus brevispinus		
	Sclerocactus cloverae		
	Sclerocactus erectocentrus		
	Sclerocactus glaucus		
	Sclerocactus mariposensis		
	Sclerocactus mesae-verdae		
	Sclerocactus nyensis		
	Sclerocactus papyracanthus		
	Sclerocactus pubispinus		

	Appendices I	II	III
	Sclerocactus sileri<br>***Sclerocactus wetlandicus***<br>***Sclerocactus wrightiae***<br>***Strombocactus* spp.**<br>***Turbinicarpus* spp.**<br>***Uebelmannia* spp.**		
CARYOCARACEAE Ajo			
		Caryocar costaricense #4	
COMPOSITAE (ASTERACEAE) Kuth			
	Saussurea costus		***Crassothonna clavifolia*** (South Africa)<br>***Othonna armiana*** (South Africa)<br>***Othonna cacalioides*** (South Africa)<br>***Othonna euphorbioides*** (South Africa)<br>***Othonna retrorsa*** (South Africa)
CRASSULACEAE Roseroot			
		***Rhodiola* spp.**#2	***Tylecodon bodleyae*** (South Africa)<br>***Tylecodon nolteei*** (South Africa)<br>***Tylecodon reticulatus*** (South Africa)

#4 All parts and derivatives, except:

a) seeds (including seedpods of Orchidaceae), spores and pollen (including pollinia). The exemption does not apply to seeds from Cactaceae spp. exported from Mexico, and to seeds from *Beccariophoenix madagascariensis* and *Dypsis decaryi* exported from Madagascar;
b) seedling or tissue cultures obtained *in vitro* transported in sterile containers;
c) cut flowers of artificially propagated plants;
d) fruits, and parts and derivatives thereof, of naturalized or artificially propagated plants of the genus *Vanilla* (Orchidaceae) and of the family Cactaceae;
e) stems, flowers, and parts and derivatives thereof, of naturalized or artificially propagated plants of the genera *Opuntia* subgenus *Opuntia* and *Selenicereus* (Cactaceae);
f) finished products of *Aloe ferox* and *Euphorbia antisyphilitica* packaged and ready for retail trade; and
g) finished products derived from artificial propagation, packaged and ready for retail trade of cosmetics containing parts and derivatives of *Bletilla striata, Cycnoches cooperi, Gastrodia elata, Phalaenopsis amabilis* or *Phalaenopsis lobbii*.

#2 All parts and derivatives except:

a) seeds and pollen; and
b) finished products packaged and ready for retail trade.

	Appendices I	II	III
CUCURBITACEAE Melons, gourds, cucurbits			
		Zygosicyos pubescens<br>***Zygosicyos tripartitus***	
CUPRESSACEAE Cypresses			
	Fitzroya cupressoides<br>***Pilgerodendron uviferum***	***Widdringtonia whytei***	
CYATHEACEAE Tree-ferns			
		***Cyathea* spp.** #4	
CYCADACEAE Cycads			
	Cycas beddomei	**CYCADACEAE spp.** #4 (Except the species included in Appendix **I**)	
DICKSONIACEAE Tree-ferns			
		Cibotium barometz #4<br>***Dicksonia* spp.** #4 (Only the populations of the Americas; no other population is included in the Appendices)	
DIDIEREACEAE Alluaudias, didiereas			
		**DIDIEREACEAE spp.** #4	
DIOSCOREACEAE Elephant’s foot			
		Dioscorea deltoidea #4	
DROSERACEAE Venus flytrap			
		Dionaea muscipula #4	

#4 All parts and derivatives, except:

a) seeds (including seedpods of Orchidaceae), spores and pollen (including pollinia). The exemption does not apply to seeds from Cactaceae spp. exported from Mexico, and to seeds from *Beccariophoenix madagascariensis* and *Dypsis decaryi* exported from Madagascar;
b) seedling or tissue cultures obtained *in vitro* transported in sterile containers;
c) cut flowers of artificially propagated plants;
d) fruits, and parts and derivatives thereof, of naturalized or artificially propagated plants of the genus *Vanilla* (Orchidaceae) and of the family Cactaceae;
e) stems, flowers, and parts and derivatives thereof, of naturalized or artificially propagated plants of the genera *Opuntia* subgenus *Opuntia* and *Selenicereus* (Cactaceae);
f) finished products of *Aloe ferox* and *Euphorbia antisyphilitica* packaged and ready for retail trade; and
g) finished products derived from artificial propagation, packaged and ready for retail trade of cosmetics containing parts and derivatives of *Bletilla striata, Cycnoches cooperi, Gastrodia elata, Phalaenopsis amabilis* or *Phalaenopsis lobbii*.

	Appendices I	II	III
EBENACEAE Ebonies			
		***Diospyros* spp.** [#5] (Only the populations of Madagascar; no other population is included in the Appendices)	
EUPHORBIACEAE Spurges			
	Euphorbia ambovombensis<br>***Euphorbia capsaintemariensis***<br>***Euphorbia cremersii*** {Includes the **forma *viridifolia*** and the **var. *rakotozafyi***}<br>***Euphorbia cylindrifolia*** {Includes the **ssp. *tuberifera***}<br>***Euphorbia decaryi*** {Includes the **vars. *ampanihyensis, robinsonii*** and ***spirosticha***}<br>***Euphorbia francoisii***<br>***Euphorbia moratii*** {Includes the **vars. *antsingiensis*, *bemarahensis*** and ***multiflora***}<br>***Euphorbia parvicyathophora***	***Euphorbia* spp.**[P2] [#4] (Succulent species only, except the species included in Appendix I and *Euphorbia misera* which is not included in the Appendices)	

[#5] Logs, sawn wood and veneer sheets.

[P2] Artificially propagated specimens of cultivars of *Euphorbia trigona*, artificially propagated specimens of crested, fan-shaped or colour mutants of *Euphorbia lactea*, when grafted on artificially propagated root stock of *Euphorbia neriifolia*, and artificially propagated specimens of cultivars of *Euphorbia* 'Milii' when they are traded in shipments of 100 or more plants and readily recognizable as artificially propagated specimens, are not subject to the provisions of the Convention.

[#4] All parts and derivatives, except:
a) seeds (including seedpods of Orchidaceae), spores and pollen (including pollinia). The exemption does not apply to seeds from Cactaceae spp. exported from Mexico, and to seeds from *Beccariophoenix madagascariensis* and *Dypsis decaryi* exported from Madagascar;
b) seedling or tissue cultures obtained *in vitro* transported in sterile containers;
c) cut flowers of artificially propagated plants;
d) fruits, and parts and derivatives thereof, of naturalized or artificially propagated plants of the genus *Vanilla* (Orchidaceae) and of the family Cactaceae;
e) stems, flowers, and parts and derivatives thereof, of naturalized or artificially propagated plants of the genera *Opuntia* subgenus *Opuntia* and *Selenicereus* (Cactaceae);
f) finished products of *Aloe ferox* and *Euphorbia antisyphilitica* packaged and ready for retail trade; and
g) finished products derived from artificial propagation, packaged and ready for retail trade of cosmetics containing parts and derivatives of *Bletilla striata, Cycnoches cooperi, Gastrodia elata, Phalaenopsis amabilis* or *Phalaenopsis lobbii*.

	Appendices I	II	III
	Euphorbia quartziticola<br>***Euphorbia tulearensis***		
FAGACEAE Mongolian oak			
			Quercus mongolica #5 (Russian Federation)
FOUQUIERIACEAE Ocotillos			
	Fouquieria fasciculata<br>***Fouquieria purpusii***	***Fouquieria columnaris*** #4	
GERANIACEAE Geraniums			
			Monsonia herrei (South Africa)<br>***Monsonia multifida*** (South Africa)<br>***Monsonia patersonii*** (South Africa)<br>***Pelargonium crassicaule*** (South Africa)<br>***Pelargonium triste*** (South Africa)
GNETACEAE Gnetum			
			Gnetum montanum #1 (Nepal)
JUGLANDACEAE Gavilan			
		Oreomunnea pterocarpa #4	
LAURACEAE Brazilian rosewood			

#5 Logs, sawn wood and veneer sheets.

#4 All parts and derivatives, except:
a) seeds (including seedpods of Orchidaceae), spores and pollen (including pollinia). The exemption does not apply to seeds from Cactaceae spp. exported from Mexico, and to seeds from *Beccariophoenix madagascariensis* and *Dypsis decaryi* exported from Madagascar;
b) seedling or tissue cultures obtained *in vitro* transported in sterile containers;
c) cut flowers of artificially propagated plants;
d) fruits, and parts and derivatives thereof, of naturalized or artificially propagated plants of the genus *Vanilla* (Orchidaceae) and of the family Cactaceae;
e) stems, flowers, and parts and derivatives thereof, of naturalized or artificially propagated plants of the genera *Opuntia* subgenus *Opuntia* and *Selenicereus* (Cactaceae);
f) finished products of *Aloe ferox* and *Euphorbia antisyphilitica* packaged and ready for retail trade; and
g) finished products derived from artificial propagation, packaged and ready for retail trade of cosmetics containing parts and derivatives of *Bletilla striata, Cycnoches cooperi, Gastrodia elata, Phalaenopsis amabilis* or *Phalaenopsis lobbii*.

#1 All parts and derivatives, except:
a) seeds, spores and pollen (including pollinia);
b) seedling or tissue cultures obtained *in vitro* transported in sterile containers;
c) cut flowers of artificially propagated plants; and
d) fruits, and parts and derivatives thereof, of artificially propagated plants of the genus *Vanilla*.

	Appendices I	II	III
		Aniba rosaeodora #12	
LEGUMINOSAE (FABACEAE) Afrormosia, cristobal, palisander, rosewood, sandalwood			
	Dalbergia nigra	***Afzelia* spp.**#17 (Only the African populations; no other population is included in the Appendices)<br>***Dalbergia* spp.** #15 (Except for the species included in Appendix **I**)<br>***Dipteryx* spp.**#17<br>***Guibourtia demeusei*** #15<br>***Guibourtia pellegriniana*** #15<br>***Guibourtia tessmannii*** #15<br>***Paubrasilia echinata*** #10<br>***Pericopsis elata*** #17<br>***Platymiscium parviflorum*** #4	

#12 Logs, sawn wood, veneer sheets, plywood and extracts. Finished products containing such extracts as ingredients, including fragrances, are not considered to be covered by this annotation.

#17 Logs, sawn wood, veneer sheets, plywood and transformed wood.

#15 All parts and derivatives, except:
a) Leaves, flowers, pollen, fruits, and seeds;
b) Finished products to a maximum weight of wood of the listed species of up to 10 kg per shipment;
c) Finished musical instruments, finished musical instrument parts and finished musical instrument accessories;
d) Parts and derivatives of *Dalbergia cochinchinensis*, which are covered by Annotation # 4; and
e) Parts and derivatives of *Dalbergia* spp. originating and exported from Mexico, which are covered by Annotation # 6.

#10 All parts, derivatives and finished products, except re-export of finished musical instruments, finished musical instrument accessories and finished musical instrument parts.

#4 All parts and derivatives, except:
a) seeds (including seedpods of Orchidaceae), spores and pollen (including pollinia). The exemption does not apply to seeds from Cactaceae spp. exported from Mexico, and to seeds from *Beccariophoenix madagascariensis* and *Dypsis decaryi* exported from Madagascar;
b) seedling or tissue cultures obtained *in vitro* transported in sterile containers;
c) cut flowers of artificially propagated plants;
d) fruits, and parts and derivatives thereof, of naturalized or artificially propagated plants of the genus *Vanilla* (Orchidaceae) and of the family Cactaceae;
e) stems, flowers, and parts and derivatives thereof, of naturalized or artificially propagated plants of the genera *Opuntia* subgenus *Opuntia* and *Selenicereus* (Cactaceae);
f) finished products of *Aloe ferox* and *Euphorbia antisyphilitica* packaged and ready for retail trade; and
g) finished products derived from artificial propagation, packaged and ready for retail trade of cosmetics containing parts and derivatives of *Bletilla striata, Cycnoches cooperi, Gastrodia elata, Phalaenopsis amabilis* or *Phalaenopsis lobbii*.

	Appendices I	II	III
		***Pterocarpus* spp.**[#17] (Except *Pterocarpus santalinus* which is included in Appendix II with annotation #7; only the African populations; no other population is included in the Appendices)	
		Pterocarpus santalinus [#7]	
		Senna meridionalis	
LILIACEAE Aloes			
		***Aloe* spp.** [#4] (Except the species included in Appendix I and *Aloe vera*, also referenced as *Aloe barbadensis,* which is not included in the Appendices)	
	Aloe albida		
	Aloe albiflora		
	Aloe alfredii		
	Aloe bakeri		
	Aloe bellatula		
	Aloe calcairophila		
	Aloe compressa {Includes the **vars. *paucituberculata, rugosquamosa*** and ***schistophila***}		
	Aloe delphinensis		
	Aloe descoingsii		
	Aloe fragilis		

[#17] Logs, sawn wood, veneer sheets, plywood and transformed wood.

[#7] Logs, woodchips, powder and extracts.

[#4] All parts and derivatives, except:

a) seeds (including seedpods of Orchidaceae), spores and pollen (including pollinia). The exemption does not apply to seeds from Cactaceae spp. exported from Mexico, and to seeds from *Beccariophoenix madagascariensis* and *Dypsis decaryi* exported from Madagascar;
b) seedling or tissue cultures obtained *in vitro* transported in sterile containers;
c) cut flowers of artificially propagated plants;
d) fruits, and parts and derivatives thereof, of naturalized or artificially propagated plants of the genus *Vanilla* (Orchidaceae) and of the family Cactaceae;
e) stems, flowers, and parts and derivatives thereof, of naturalized or artificially propagated plants of the genera *Opuntia* subgenus *Opuntia* and *Selenicereus* (Cactaceae);
f) finished products of *Aloe ferox* and *Euphorbia antisyphilitica* packaged and ready for retail trade; and
g) finished products derived from artificial propagation, packaged and ready for retail trade of cosmetics containing parts and derivatives of *Bletilla striata, Cycnoches cooperi, Gastrodia elata, Phalaenopsis amabilis* or *Phalaenopsis lobbii*.

	Appendices<br>I	 II	 III
	Aloe haworthioides {Includes the var. ***aurantiaca***}<br>***Aloe helenae***<br>***Aloe laeta*** {Includes the var. ***maniaensis***}<br>***Aloe parallelifolia***<br>***Aloe parvula***<br>***Aloe pillansii***<br>***Aloe polyphylla***<br>***Aloe rauhii***<br>***Aloe suzannae***<br>***Aloe versicolor***<br>***Aloe vossii***		
MAGNOLIACEAE Magnolias			
			Magnolia liliifera* var. *obovata #1 (Nepal)
MALVACEAE Baobab			
		Adansonia grandidieri #16	
MELIACEAE Mahoganies, West Indian cedar			
		Cedrela* spp.** #6 (Only the populations of the Neotropics; no other population is included in the Appendices)<br>Khaya* spp.** #17 (Only the African populations; no other population is included in the Appendices)	

#1 All parts and derivatives, except:
a) seeds, spores and pollen (including pollinia);
b) seedling or tissue cultures obtained *in vitro* transported in sterile containers;
c) cut flowers of artificially propagated plants; and
d) fruits, and parts and derivatives thereof, of artificially propagated plants of the genus *Vanilla*.

#16 Seeds, fruits and oils.

#6 Logs, sawn wood, veneer sheets and plywood.

#17 Logs, sawn wood, veneer sheets, plywood and transformed wood.

	Appendices I	II	III
		Swietenia humilis #4<br>***Swietenia macrophylla*** #6 (Only the populations of the Neotropics; no other population is included in the Appendices)<br>***Swietenia mahagoni*** #5	
NEPENTHACEAE Pitcher-plants (Old World)			
	Nepenthes khasiana<br>***Nepenthes rajah***	***Nepenthes* spp.** #4 (Except the species included in Appendix I)	
OLEACEAE Machurian ash			
			Fraxinus mandshurica #5 (Russian Federation)

#4 All parts and derivatives, except:
a) seeds (including seedpods of Orchidaceae), spores and pollen (including pollinia). The exemption does not apply to seeds from Cactaceae spp. exported from Mexico, and to seeds from *Beccariophoenix madagascariensis* and *Dypsis decaryi* exported from Madagascar;
b) seedling or tissue cultures obtained *in vitro* transported in sterile containers;
c) cut flowers of artificially propagated plants;
d) fruits, and parts and derivatives thereof, of naturalized or artificially propagated plants of the genus *Vanilla* (Orchidaceae) and of the family Cactaceae;
e) stems, flowers, and parts and derivatives thereof, of naturalized or artificially propagated plants of the genera *Opuntia* subgenus *Opuntia* and *Selenicereus* (Cactaceae);
f) finished products of *Aloe ferox* and *Euphorbia antisyphilitica* packaged and ready for retail trade; and
g) finished products derived from artificial propagation, packaged and ready for retail trade of cosmetics containing parts and derivatives of *Bletilla striata, Cycnoches cooperi, Gastrodia elata, Phalaenopsis amabilis* or *Phalaenopsis lobbii*.

#6 Logs, sawn wood, veneer sheets and plywood.

#5 Logs, sawn wood and veneer sheets.

	Appendices I	II	III
ORCHIDACEAE Orchids			
	Aerangis ellisii P4<br>***Cattleya jongheana*** P4<br>***Cattleya lobata*** P4<br>***Dendrobium cruentum*** P4<br>***Mexipedium xerophyticum*** P4<br>***Paphiopedilum* spp.** P4<br>***Peristeria elata*** P4<br>***Phragmipedium* spp.** P4<br>***Renanthera imschootiana*** P4	**ORCHIDACEAE spp.** P3 #4 (Except the species included in Appendix I)	
OROBANCHACEAE Desert cistanche			
		Cistanche deserticola #4	
PALMAE (ARECACEAE) Palms			
		Beccariophoenix madagascariensis #4<br>***Dypsis decaryi*** #4	

P3 Artificially propagated hybrids of the following genera are not subject to the provisions of the Convention, if conditions, as indicated under a) and b), are met: *Cymbidium, Dendrobium, Phalaenopsis* and *Vanda*:
a) Specimens are readily recognizable as artificially propagated and do not show any signs of having been collected in the wild such as mechanical damage or strong dehydration resulting from collection, irregular growth and heterogeneous size and shape within a taxon and shipment, algae or other epiphyllous organisms adhering to leaves, or damage by insects or other pests; and
b) i) when shipped in non-flowering state, the specimens must be traded in shipments consisting of individual containers (such as cartons, boxes, crates or individual shelves of CC-containers) each containing 20 or more plants of the same hybrid; the plants within each container must exhibit a high degree of uniformity and healthiness; and the shipment must be accompanied by documentation, such as an invoice, which clearly states the number of plants of each hybrid; or
ii) when shipped in flowering state, with at least one fully open flower per specimen, no minimum number of specimens per shipment is required but specimens must be professionally processed for commercial retail sale, e.g. labelled with printed labels or packaged with printed packages indicating the name of the hybrid and the country of final processing. This should be clearly visible and allow easy verification.

Plants not clearly qualifying for the exemption must be accompanied by appropriate CITES documents.

#4 All parts and derivatives, except:
a) seeds (including seedpods of Orchidaceae), spores and pollen (including pollinia). The exemption does not apply to seeds from Cactaceae spp. exported from Mexico, and to seeds from *Beccariophoenix madagascariensis* and *Dypsis decaryi* exported from Madagascar;
b) seedling or tissue cultures obtained *in vitro* transported in sterile containers;
c) cut flowers of artificially propagated plants;
d) fruits, and parts and derivatives thereof, of naturalized or artificially propagated plants of the genus *Vanilla* (Orchidaceae) and of the family Cactaceae;
e) stems, flowers, and parts and derivatives thereof, of naturalized or artificially propagated plants of the genera *Opuntia* subgenus *Opuntia* and *Selenicereus* (Cactaceae);
f) finished products of *Aloe ferox* and *Euphorbia antisyphilitica* packaged and ready for retail trade; and
g) finished products derived from artificial propagation, packaged and ready for retail trade of cosmetics containing parts and derivatives of *Bletilla striata, Cycnoches cooperi, Gastrodia elata, Phalaenopsis amabilis* or *Phalaenopsis lobbii*.

P4 Seedling or tissue cultures obtained *in vitro* and transported in sterile containers are not subject to the provisions of the Convention only if the specimens meet the definition of 'artificially propagated' agreed by the Conference of the Parties.

	Appendices I	II	III
	Dypsis decipiens		
		Lemurophoenix halleuxii	
			Lodoicea maldivica #13 (Seychelles)
		Marojejya darianii	
		Ravenea louvelii	
		Ravenea rivularis	
		Satranala decussilvae	
		Voanioala gerardii	
PAPAVERACEAE Himalayan poppy			
			Meconopsis regia #1 (Nepal)
PASSIFLORACEAE Passion-flowers			
		Adenia firingalavensis	
		Adenia olaboensis	
			Adenia spinosa (South Africa)
		Adenia subsessilifolia	
PEDALIACEAE Sesames			
		Uncarina grandidieri	
		Uncarina stellulifera	
PINACEAE Firs and pines			
	Abies guatemalensis		
			Pinus koraiensis #5 (Russian Federation)
PODOCARPACEAE Podocarps			
			Podocarpus neriifolius #1 (Nepal)
	Podocarpus parlatorei		

#13 The kernel (also known as 'endosperm', 'pulp' or 'copra') and any derivatives thereof, except finished products packaged and ready for retail trade.

#1 All parts and derivatives, except:
a) seeds, spores and pollen (including pollinia);
b) seedling or tissue cultures obtained *in vitro* transported in sterile containers;
c) cut flowers of artificially propagated plants; and
d) fruits, and parts and derivatives thereof, of artificially propagated plants of the genus *Vanilla*.

#5 Logs, sawn wood and veneer sheets.

	Appendices<br>I	 II	 III
PORTULACACEAE Lewisias, portulacas, purslanes			
		Anacampseros* spp.** #4<br>Avonia* spp.** #4<br>***Lewisia serrata*** #4	***Portulacaria pygmaea*** (South Africa)
PRIMULACEAE Cyclamens			
		***Cyclamen* spp.** P5 #4	
RANUNCULACEAE Yellow adonis, yellow root			
		Adonis vernalis #2<br>***Hydrastis canadensis*** #8	
ROSACEAE African cherry			
		Prunus africana #4	
RUBIACEAE Ayugue			
	Balmea stormiae		

#4 All parts and derivatives, except:
a) seeds (including seedpods of Orchidaceae), spores and pollen (including pollinia). The exemption does not apply to seeds from Cactaceae spp. exported from Mexico, and to seeds from *Beccariophoenix madagascariensis* and *Dypsis decaryi* exported from Madagascar;
b) seedling or tissue cultures obtained *in vitro* transported in sterile containers;
c) cut flowers of artificially propagated plants;
d) fruits, and parts and derivatives thereof, of naturalized or artificially propagated plants of the genus *Vanilla* (Orchidaceae) and of the family Cactaceae;
e) stems, flowers, and parts and derivatives thereof, of naturalized or artificially propagated plants of the genera *Opuntia* subgenus *Opuntia* and *Selenicereus* (Cactaceae);
f) finished products of *Aloe ferox* and *Euphorbia antisyphilitica* packaged and ready for retail trade; and
g) finished products derived from artificial propagation, packaged and ready for retail trade of cosmetics containing parts and derivatives of *Bletilla striata, Cycnoches cooperi, Gastrodia elata, Phalaenopsis amabilis* or *Phalaenopsis lobbii*.

P5 Artificially propagated specimens of cultivars of *Cyclamen persicum* are not subject to the provisions of the Convention. However, the exemption does not apply to such specimens traded as dormant tubers.

#2 All parts and derivatives except:
a) seeds and pollen; and
b) finished products packaged and ready for retail trade.

#8 Underground parts (i.e. roots, rhizomes): whole, parts and powdered.

	Appendices I	II	III
SANTALACEAE African sandalwood			
		Osyris lanceolata #2 (Only the populations of Burundi, Ethiopia, Kenya, Rwanda, Uganda and the United Republic of Tanzania; no other population is included in the Appendices)	
SARRACENIACEAE Pitcher-plants (New World)			
	Sarracenia oreophila<br>***Sarracenia rubra* ssp. *alabamensis***<br>***Sarracenia rubra* ssp. *jonesii***	***Sarracenia* spp.** #4 (Except the species included in Appendix I)	
SCROPHULARIACEAE Kutki			
		Picrorhiza kurrooa #2 {Excludes *Picrorhiza scrophulariiflora*}	
STANGERIACEAE Stangerias			
	Stangeria eriopus	***Bowenia* spp.** #4	

#2 All parts and derivatives except:
a) seeds and pollen; and
b) finished products packaged and ready for retail trade.

#4 All parts and derivatives, except:
a) seeds (including seedpods of Orchidaceae), spores and pollen (including pollinia). The exemption does not apply to seeds from Cactaceae spp. exported from Mexico, and to seeds from *Beccariophoenix madagascariensis* and *Dypsis decaryi* exported from Madagascar;
b) seedling or tissue cultures obtained *in vitro* transported in sterile containers;
c) cut flowers of artificially propagated plants;
d) fruits, and parts and derivatives thereof, of naturalized or artificially propagated plants of the genus *Vanilla* (Orchidaceae) and of the family Cactaceae;
e) stems, flowers, and parts and derivatives thereof, of naturalized or artificially propagated plants of the genera *Opuntia* subgenus *Opuntia* and *Selenicereus* (Cactaceae);
f) finished products of *Aloe ferox* and *Euphorbia antisyphilitica* packaged and ready for retail trade; and
g) finished products derived from artificial propagation, packaged and ready for retail trade of cosmetics containing parts and derivatives of *Bletilla striata, Cycnoches cooperi, Gastrodia elata, Phalaenopsis amabilis* or *Phalaenopsis lobbii*.

	Appendices I	II	III
TAXACEAE Yews			
		Taxus chinensis #2 {Includes infraspecific taxa of this species}<br>***Taxus cuspidata*** P6 #2 {Includes infraspecific taxa of this species}<br>***Taxus fuana*** #2 {Includes infraspecific taxa of this species}<br>***Taxus sumatrana*** #2 {Includes infraspecific taxa of this species}<br>***Taxus wallichiana*** #2	
THYMELAEACEAE (AQUILARIACEAE) Agarwood, ramin			
		Aquilaria* spp.** #14<br>Gonystylus* spp.** #4<br>***Gyrinops* spp.** #14	

#2 All parts and derivatives except:
a) seeds and pollen; and
b) finished products packaged and ready for retail trade.

P6 Artificially propagated hybrids and cultivars of *Taxus cuspidata,* live, in pots or other small containers, each consignment being accompanied by a label or document stating the name of the taxon or taxa and the text 'artificially propagated', are not subject to the provisions of the Convention.

#14 All parts and derivatives except:
a) seeds and pollen;
b) seedling or tissue cultures obtained *in vitro* transported in sterile containers;
c) fruits;
d) leaves;
e) exhausted agarwood powder, including compressed powder in all shapes; and
f) finished products packaged and ready for retail trade, this exemption does not apply to wood chips, beads, prayer beads and carvings.

#4 All parts and derivatives, except:
a) seeds (including seedpods of Orchidaceae), spores and pollen (including pollinia). The exemption does not apply to seeds from Cactaceae spp. exported from Mexico, and to seeds from *Beccariophoenix madagascariensis* and *Dypsis decaryi* exported from Madagascar;
b) seedling or tissue cultures obtained *in vitro* transported in sterile containers;
c) cut flowers of artificially propagated plants;
d) fruits, and parts and derivatives thereof, of naturalized or artificially propagated plants of the genus *Vanilla* (Orchidaceae) and of the family Cactaceae;
e) stems, flowers, and parts and derivatives thereof, of naturalized or artificially propagated plants of the genera *Opuntia* subgenus *Opuntia* and *Selenicereus* (Cactaceae);
f) finished products of *Aloe ferox* and *Euphorbia antisyphilitica* packaged and ready for retail trade; and
g) finished products derived from artificial propagation, packaged and ready for retail trade of cosmetics containing parts and derivatives of *Bletilla striata, Cycnoches cooperi, Gastrodia elata, Phalaenopsis amabilis* or *Phalaenopsis lobbii*.

	Appendices I	II	III
TROCHODENDRACEAE (TETRACENTRACEAE) Tetracentron			
			Tetracentron sinense #1 (Nepal)
VALERIANACEAE Himalayan spikenard			
		Nardostachys grandiflora #2	
VITACEAE Grapes			
		Cyphostemma elephantopus<br>***Cyphostemma laza***<br>***Cyphostemma montagnacii***	
WELWITSCHIACEAE Welwitschia			
		Welwitschia mirabilis #4	
ZAMIACEAE Cycads			
	Ceratozamia* spp.**<br>Encephalartos* spp.**<br>***Microcycas calocoma***<br>***Zamia restrepoi***	**ZAMIACEAE spp.** #4 (Except the species included in Appendix I)	

#1 All parts and derivatives, except:
a) seeds, spores and pollen (including pollinia);
b) seedling or tissue cultures obtained *in vitro* transported in sterile containers;
c) cut flowers of artificially propagated plants; and
d) fruits, and parts and derivatives thereof, of artificially propagated plants of the genus *Vanilla*.

#2 All parts and derivatives except:
a) seeds and pollen; and
b) finished products packaged and ready for retail trade.

#4 All parts and derivatives, except:
a) seeds (including seedpods of Orchidaceae), spores and pollen (including pollinia). The exemption does not apply to seeds from Cactaceae spp. exported from Mexico, and to seeds from *Beccariophoenix madagascariensis* and *Dypsis decaryi* exported from Madagascar;
b) seedling or tissue cultures obtained *in vitro* transported in sterile containers;
c) cut flowers of artificially propagated plants;
d) fruits, and parts and derivatives thereof, of naturalized or artificially propagated plants of the genus *Vanilla* (Orchidaceae) and of the family Cactaceae;
e) stems, flowers, and parts and derivatives thereof, of naturalized or artificially propagated plants of the genera *Opuntia* subgenus *Opuntia* and *Selenicereus* (Cactaceae);
f) finished products of *Aloe ferox* and *Euphorbia antisyphilitica* packaged and ready for retail trade; and
g) finished products derived from artificial propagation, packaged and ready for retail trade of cosmetics containing parts and derivatives of *Bletilla striata, Cycnoches cooperi, Gastrodia elata, Phalaenopsis amabilis* or *Phalaenopsis lobbii*.

	Appendices I	II	III
ZINGIBERACEAE Ginger lily, Natal ginger			
		Hedychium philippinense #4<br>***Siphonochilus aethiopicus*** (Only the populations of Eswatini, Mozambique, South Africa and Zimbabwe; no other population is included in the Appendices)	
ZYGOPHYLLACEAE Lignum-vitae			
		Bulnesia sarmientoi #11<br>***Guaiacum* spp.** #2	

#4 All parts and derivatives, except:

a) seeds (including seedpods of Orchidaceae), spores and pollen (including pollinia). The exemption does not apply to seeds from Cactaceae spp. exported from Mexico, and to seeds from *Beccariophoenix madagascariensis* and *Dypsis decaryi* exported from Madagascar;
b) seedling or tissue cultures obtained *in vitro* transported in sterile containers;
c) cut flowers of artificially propagated plants;
d) fruits, and parts and derivatives thereof, of naturalized or artificially propagated plants of the genus *Vanilla* (Orchidaceae) and of the family Cactaceae;
e) stems, flowers, and parts and derivatives thereof, of naturalized or artificially propagated plants of the genera *Opuntia* subgenus *Opuntia* and *Selenicereus* (Cactaceae);
f) finished products of *Aloe ferox* and *Euphorbia antisyphilitica* packaged and ready for retail trade; and
g) finished products derived from artificial propagation, packaged and ready for retail trade of cosmetics containing parts and derivatives of *Bletilla striata, Cycnoches cooperi, Gastrodia elata, Phalaenopsis amabilis* or *Phalaenopsis lobbii*.

#11 Logs, sawn wood, veneer sheets, plywood, powder and extracts. Finished products containing such extracts as ingredients, including fragrances, are not considered to be covered by this annotation.

#2 All parts and derivatives except:

a) seeds and pollen; and
b) finished products packaged and ready for retail trade.
